# State-of-the-Art Review of Microcapsule Self-Repairing Concrete: Principles, Applications, Test Methods, Prospects

**DOI:** 10.3390/polym16223165

**Published:** 2024-11-13

**Authors:** Lu Jiang, Mingli Wu, Fei Du, Dongdong Chen, Lihua Xiao, Wei Chen, Wei Du, Qingjun Ding

**Affiliations:** 1 Hubei Provincial Engineering Research Center of Industrial Detonator Intelligent Assembly, Wuhan Textile University, Wuhan 430073, China; jl0107023@163.com; 2 School of Materials Science and Engineering, Wuhan Textile University, Wuhan 430200, China; 3 College of Life Science & Technology, Huazhong University of Science and Technology, Wuhan 430074, China; d202280823@hust.edu.cn; 4 Central & Southern China Municipal Engineering Design and Research Institute Co., Ltd., Wuhan 430010, China; dophee@126.com; 5 School of Civil and Hydraulic Engineering, Huazhong University of Science and Technology, Wuhan 430074, China; 6 State Key Laboratory of Silicate Materials for Architectures, Wuhan University of Technology, Wuhan 430070, China; chendongdong0714@163.com (D.C.); dingqj@whut.edu.cn (Q.D.); 7 Department of Automotive Engineering, Guizhou Communications Polytechnic University, Guiyang 551400, China; xiaolihua@git.edu.cn; 8 School of Materials Science and Engineering, Central South University, Changsha 410083, China

**Keywords:** cement-based materials, self-repairing, crack, microcapsule, nanomaterials, durability

## Abstract

Cement-based materials are widely used in construction worldwide, but they are vulnerable to environmental stressors and thermal fluctuations, leading to the formation of internal cracks that compromise structural integrity and durability. Traditional repair methods such as surface coatings, grouting, and groove filling are often costly and labor-intensive. In response, self-repairing technologies for cement-based materials have emerged as an innovative and promising solution, offering the potential to significantly extend the lifespan of structures and reduce maintenance costs. A particularly novel approach is the development of microcapsule-based self-repairing concrete. In this system, repair agents are encapsulated within microcapsules and combined with curing agents in the concrete matrix. When cracks form, the microcapsules rupture, releasing the repair agents to autonomously heal the damage. This self-repairing mechanism is characterized by its high efficiency, durability, environmental sustainability, and versatility, making it a promising alternative to traditional repair methods. Recent research has focused on the development of microcapsules with various core materials, such as TDI (toluene diisocyanate), IPDI (isophorone diisocyanate), or epoxy resin, as well as composite shell materials including paraffin wax, PE (polyethylene) wax, nano-SiO_2_, and nano-CaCO_3_. A novel advancement in this area involves the enhancement of microcapsules through the incorporation of magnetic nanomaterials into the shell, providing new possibilities for self-repairing systems that address cracks in cement-based materials.

## 1. Introduction

Cement-based materials are widely acclaimed for their exceptional compressive strength and durability, making them the preferred choice for various construction applications. However, these materials are prone to cracking during use, which poses a significant threat to structural ability, longevity, and aesthetic appeal. If left unaddressed, cracks can expand over time, facilitating the ingress of corrosive ions and ultimately leading to structural deterioration. The lack of regular maintenance exacerbates this problem, underscoring the importance of proactive measures. A report published by the American Society of Civil Engineers (ASCE) highlights substantial financial requirements for infrastructure maintenance in the United States (USD 2.2 trillion) and Asian nations (USD 2 trillion) over the next five years [1]. Traditional methods such as surface coating, grouting, and trench filling, used for inspecting, maintaining, and repairing concrete structures, present significant drawbacks, including production interruptions and economic losses [2]. Studies on the self-repairing properties of cement-based materials encompass various methods and strategies. Research on self-repairing cement-based materials dates to the 1990s, initially focusing on polymer-modified concrete. Over the years, the research scope has progressively broadened to include various forms of fiber-reinforced self-repairing concretes. In 2007, the IOP program in the Netherlands and the first International Conference on Self-repairing Materials significantly contributed to the global recognition and interest in self-repairing concrete, attracting research groups worldwide [3]. Recently, scientists have investigated self-repairing mechanisms that rely on biomaterials, such as microbial-induced calcification processes. Additionally, researchers have explored self-repairing techniques utilizing shape-memory alloys and principles inspired by nature [4,5,6]. Exploring these materials is a crucial aspect of the cutting-edge field of materials science and presents potential methods for constructing durable and environmentally friendly concrete structures [7].

Despite the promising theoretical and experimental advancements, the practical implementation of self-repairing concrete encounters several significant challenges. These include issues related to reliability and stability, cost and economic feasibility, material selection and optimization, environmental influences, standardization and specification development, and the continuous monitoring and assessment of long-term performance. Chrysoula et al. [3] developed polymeric microcapsules containing a liquid sodium silicate core, which were integrated into reinforced concrete elements during full-scale field trials, confirming the potential of microcapsule-based technology for delivering healing capabilities in new structures. Additionally, Yang et al. [4] employed interface self-assembly and sol–gel methods to synthesize microcapsules using methyl methacrylate monomers as the repair agent, which were then dispersed into cement mortar, resulting in a significant reduction in specimen permeability. Li et al. [5] used urea-formaldehyde resin to coat epoxy resin to produce microcapsules, which were found to enhance the self-healing efficiency in terms of crack width and depth reduction as well as permeability and strength recovery in cracked mortar specimens. These studies illustrate the diverse applications of microcapsules in enhancing the self-repair capabilities of concrete in various structural applications. While self-repairing concrete has demonstrated potential in controlled laboratory settings, its effectiveness in real-world applications remains to be fully validated. The unique adaptations required for various self-repair mechanisms and the environmental influences further complicate the selection and optimization of suitable materials. Environmental factors such as temperature, humidity, and chemical exposure can significantly impact the efficacy of self-repairing concrete. There is also a pressing need for standardization and specification development to guide the use of self-repairing concrete in practical applications. Continuous monitoring and rigorous assessment of its long-term performance are essential to ensure its effectiveness and reliability in real-world conditions [6,7,8,9,10,11].

**Figure 1:**
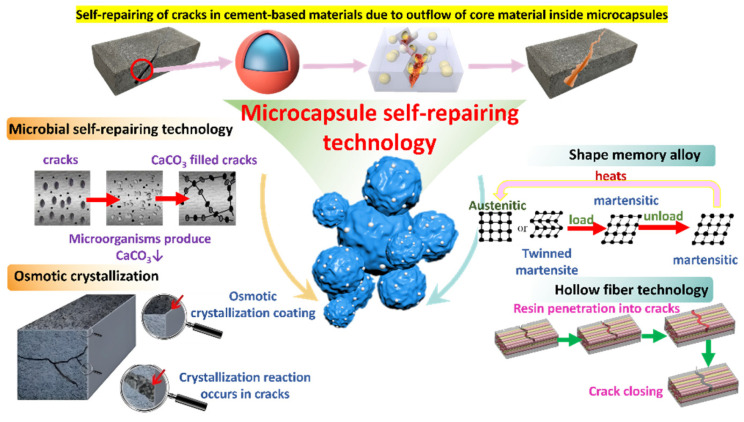
Types of self-repairing of cement-based materials.

Subsequent investigations are expected to prioritize the feasibility and cost-effectiveness of self-repairing concrete while delving into novel technologies and materials to enhance the efficiency and eco-friendliness of self-repairing processes. After elucidating the fundamental principles of five self-repairing technologies (as depicted in Figure 1), this review consolidates the results of the application accomplishments of each technology. It furnishes an overview of their current advancements and limitations, providing insights into the prospects for future development.

### 1.1. Harm of Cracks to Cement-Based Materials

**Figure 2:**
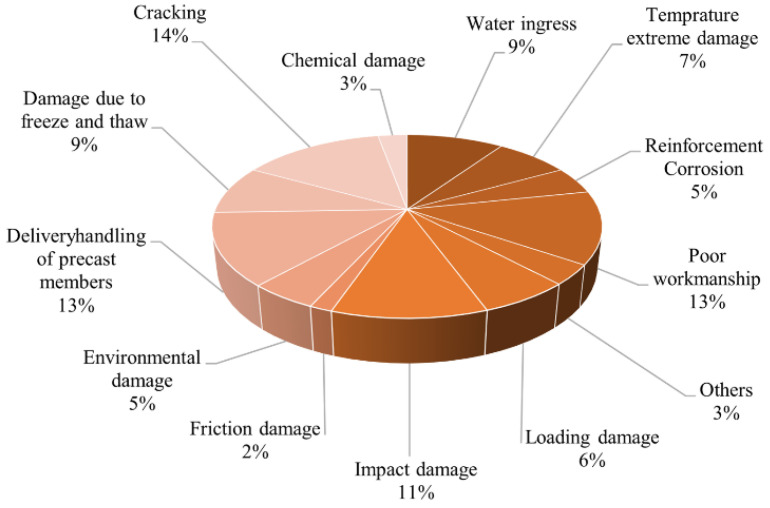
Main causes of deterioration/damage of cement-based materials.

**Figure 3:**
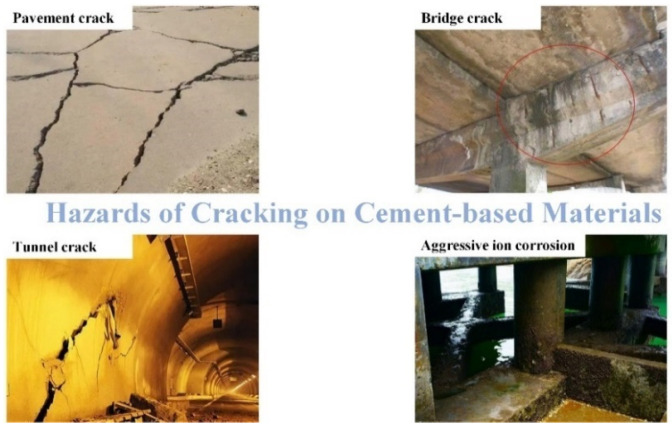
The harm of cracks to cement-based materials.

**Figure 4:**
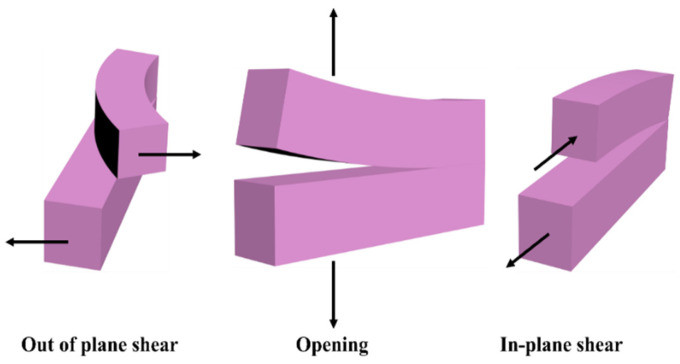
Cracking mode of structure.

The initial cracks observed in cement-based materials tend to progressively widen under the influence of loads and stress, eventually leading to the formation of visible cracks. Figure 2 visually represents the key factors contributing to the deterioration and damage of cement-based materials. Marsavina et al. [12] elucidated that the failure mechanism of these materials is intricately linked to the initiation and propagation of cracks. These cracks serve as conduits for infiltrating corrosive ions (such as Cl^−^, SO_4_^2−^, CO_3_^2−^, etc.) into the interior of cement-based materials. This infiltration initiates the corrosion of the passivation film surrounding internal steel reinforcement, resulting in rust expansion, increased tensile stress within the structure, and gradual degradation and detachment of the cement-based material. This deterioration not only affects the aesthetic appearance but also compromises the structural integrity, as illustrated in Figure 3. The propagation of cracks is influenced by three primary modes: the opening mode (where the crack surfaces move perpendicular to the crack plane), the translational sliding mode (where the crack surfaces move within the crack plane and perpendicular to the crack front edge direction), and the rotational sliding mode (where the crack surfaces move within the crack plane and parallel to the crack front edge direction) [13], as depicted in Figure 4.

Although newly formed microcracks do not immediately compromise the integrity of concrete structures, they facilitate the ingress of harmful substances such as water and chloride ions, thereby affecting the durability of buildings [14]. Therefore, enhancing the damage resistance of concrete structures is crucial for ensuring structural safety and optimizing resource utilization efficiently.

### 1.2. Traditional Repair Methods

Traditional methods for repairing cracks in cement-based materials such as surface coatings, grouting, and groove filling are employed to protect concrete structures under specific environmental conditions, especially where structural protection is inadequate, or cracks appear in the substrate. These methods aim to shield the surface of uncracked concrete, thereby prolonging the lifespan of structure [15]. Despite the favorable physical and mechanical properties of coatings, their application in similar scenarios has often been unsuccessful. This is due to the dual requirement that coatings must not only resist substances present in corrosive environments but also withstand stresses induced by the emergence of cracks and variations in crack width [16]. Thus, selecting appropriate coatings to protect reinforced concrete structures is a complex task. It necessitates careful consideration of the root causes of structural degradation, operational conditions, applied loads, and significant variability in the grade and quality of the concrete used [17,18].

Grouting repair is a widely utilized technique for addressing concrete cracks, with its efficacy influenced by factors such as the choice of grouting materials, underlying grouting repair theory, and associated technology [19]. This method is proficient in sealing cracks, thereby mitigating the risk of steel corrosion due to groundwater infiltration [20]. Chemical agents, including polyurethane and epoxy resins, are commonly employed for crack treatment; however, these materials are prone to aging and possess mechanical properties distinct from those of concrete. The typical crack repair process involves drilling and injecting grouting materials, with the angle and depth of drilling being critical determinants of the diffusion theory. Despite its importance, the diffusion mechanism within cracks remains poorly understood, which may adversely affect the quality of crack repair.

When crack widths exceed 0.3 mm, conventional grouting methods may offer only partial remediation. In such cases, the groove-filling method is employed, which entails filling the crack with a suitable repair material. This process begins with a comprehensive inspection and diagnosis of the concrete structure, followed by meticulous surface cleaning to ensure robust adhesion. However, this method presents several limitations, including its complexity, time-consuming nature, limited suitability for small and shallow cracks, inapplicability to dynamic cracks, and dependency on the quality of the repair materials used. Consequently, alternative approaches are necessary for addressing wide or deep cracks, as substandard filling materials can lead to ineffective repairs and necessitate frequent maintenance.

Traditional methods for repairing cracks in cement-based materials exhibit several significant shortcomings. Firstly, these methods often result in short-term repair outcomes, with crack issues persisting and necessitating frequent maintenance. Secondly, the high costs and extended project downtimes associated with these methods lead to production losses. Additionally, conventional restoration techniques may have adverse environmental impacts. They are ineffective at accommodating dynamically evolving cracks and are unable to adapt to structural deformations. These limitations highlight the necessity for more flexible and durable repair solutions.

### 1.3. Self-Repairing Methods

Self-repairing technology represents a significant advancement over traditional methods for addressing concrete cracks. Inspired by biological tissues, self-repairing concrete systems release substances to autonomously mend damage, thereby mimicking natural repair mechanisms to respond to cracks through self-sealing and repair. For internal structural cracks that are difficult to detect, these automatic repair mechanisms can effectively prevent crack propagation and mitigate associated losses in a timely manner. Numerous experiments and studies have introduced innovative self-repairing mechanisms and methods for cement-based materials, including shape-memory alloys and osmotic crystallization as well as intrinsic self-repairing, microbial self-repairing, hollow fiber self-repairing, and microcapsule self-repairing materials [21]. Recent research on the self-repairing performance of cement-based materials has garnered considerable attention, focusing on (1) elucidating the principles and characteristics of self-repairing technologies; (2) comparing the applications, advantages, and limitations of each repair method; and (3) developing techniques for crack detection and evaluating repair effectiveness. This review synthesizes and analyzes the current research findings in these areas and outlines future research directions, aiming to provide a comprehensive reference for ongoing studies in self-repair technology for cement-based materials.

## 2. Self-Repairing Technology

Cement-based materials, while prone to cracking, have the potential for self-repair under specific conditions. By leveraging and optimizing the intrinsic self-repair mechanisms of these materials, it is possible to engineer intelligent cement-based materials with self-diagnosis and self-repair capabilities. However, manual control during the crack repair process in self-repairing concrete can be challenging, often leading to residual small particles and impurities at the concrete interface within the crack area. This can result in the following issues: the presence of impurities such as uncleaned concrete fines and loose layers at the bonding interface; the complexity of the bonding interface making it difficult to effectively control the thickness and uniformity of the internal adhesive; incomplete evaporation of the solvent in the adhesive layer; and inadequate curing pressure potentially affecting the repair effectiveness. To ensure satisfactory repair outcomes, the repair materials in self-repairing concrete must meet the following seven performance criteria: (1) simple curing conditions and a moderate curing rate; (2) a bonding process that is simple and reliable without requiring specific constraints such as pressure; (3) moderate polarity and molecular weight to withstand various forces post repair, necessitating high bonding strength and cured strength to prevent secondary cracking; (4) moderate viscosity and excellent fluidity; (5) stable chemical properties and an extended shelf life and resistance to environmental factors and aging, with good freeze–thaw resistance to ensure prolonged concrete service life; (6) ease of transportation and storage, environmentally friendly, and non-toxic or low in toxicity; and (7) economical pricing, suitable for large-scale production.

### 2.1. Intrinsic Self-Repairing

**Figure 5:**
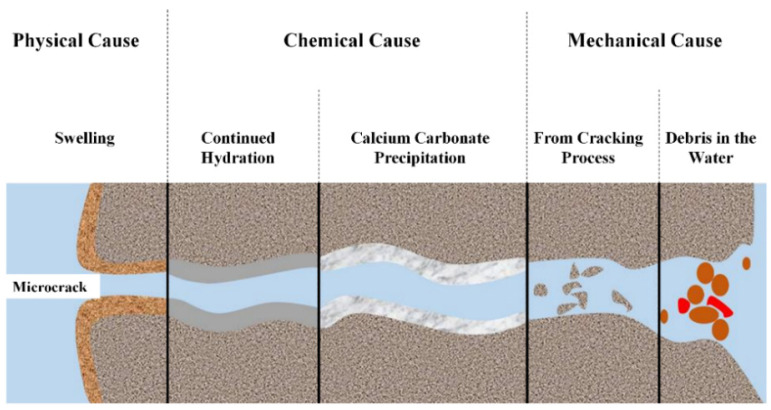
The mechanisms of autogenous repairing for concrete [23].

Intrinsic self-repairing is a fundamental property of cement-based materials, characterized by the partial or complete filling of microcracks within the cement matrix through various physical and chemical processes [22]. Extensive research by numerous scholars has elucidated several reactions occurring during the intrinsic self-repairing process (Figure 5 [23]): (a) physical reactions such as the expansion of cement slurry on the crack surface; (b) chemical reactions including the continued hydration of un-hydrated cement particles and the formation of calcium carbonate crystals via the reaction between calcium hydroxide and carbon dioxide; and (c) mechanical actions involving the filling of cracks with sediment particles in water and the blockage of cracks with concrete peelings [24].

Reinhardt et al. [24] investigated the correlation between the rate of self-repair in concrete and temperature and crack width through experimental trials. They subjected experimental samples to temperatures of 20 °C, 50 °C, and 80 °C for repairing tests, observing that narrower cracks exhibited faster repairing rates, and higher temperatures accelerated crack repairing. Additionally, cracks smaller than 0.1 mm, with a hydraulic gradient of approximately 1 MPa/m, were deemed suitable for self-repairing and sealing. Chen et al. [25] introduced polycarboxylate superplasticizers to enhance the performance of concrete materials. Their research findings indicated that polycarboxylate-based high-efficiency water-reducing agents effectively reduced the hydration-induction period of cement–fly ash cementitious materials and significantly mitigated the heat release rate during the hydration peak. This inhibition of Ca(OH)_2_ and AFt crystallization, coupled with increased cement slurry density and reduced chemically bound water content, delayed the overall hydration process of cement-based materials. Consequently, this slowed down the adiabatic heating rate and early drying shrinkage rate of concrete while enhancing its mechanical properties and impermeability. Despite the effectiveness of this self-repairing process, which requires water and air exposure, its progression is slow and offers limited utility in practical engineering applications. Thus, it should be combined with a variety of repair techniques to enhance the efficiency and speed of self-repairing concrete.

### 2.2. Microbial Self-Repairing Technology

In the early stages of oil extraction, it was discovered that microorganisms could mend cracks within the surrounding rocks of oil reservoirs, with the added observation that these microorganisms continued to exhibit a mineral-binding role even post cell death [26]. Hill et al. [27] conducted a study focusing on the injection of microorganisms and nutrients into fractured rocks. The resulting microbial-induced mineralized sediments were found to effectively fill gaps between the fractured rocks, thereby enhancing the frost resistance of the rock mass. Research by Navarro et al. [28] suggested that microbial mineralization has the potential to repair cracks on damaged limestone decorative surfaces. Jonkers et al. [29] introduced the concept and initial research outcomes of using bacteria for the automatic repair of concrete in 2007, thus laying the foundation for subsequent research in microbial self-repair technology. This form of crack repair, facilitated by microorganisms, primarily stems from the environmentally friendly and compatible microbially induced calcite precipitation (MICP) technology, which has attracted global research interest.

**Figure 6:**
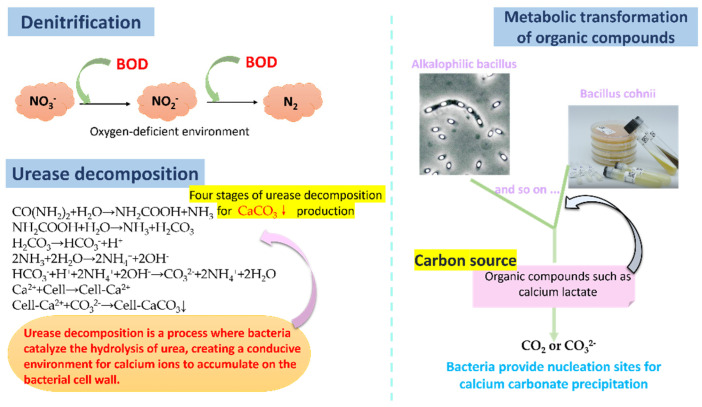
Schematic diagram of microbe-induced calcium carbonate mineralization deposition.

There exist two primary categories of deposits resulting from microbial-induced calcium carbonate mineralization: bacterial autotrophic and heterotrophic-induced calcium carbonate deposition. Bacterial autotrophic calcium carbonate deposition in cement-based materials primarily occurs through bacterial heterotrophic denitrification, urease decomposition [30], and metabolic transformation of organic matter [31]. Denitrification is a green mineralization reaction where NO_3_^−^ is used as an electron acceptor, causing calcium carbonate precipitation in a calcium-rich environment. Urease decomposition, a process where bacteria catalyze the hydrolysis of urea into ammonium and carbonate ions, results in calcium carbonate precipitation. This process is widely used in cement-based materials due to its efficient hydrolytic capability. Metabolic transformation of organic compounds also produces calcium carbonate precipitation and carbon dioxide, with organic compounds serving as calcium sources without affecting the strength of the material (as depicted in Figure 6).

**Figure 7:**
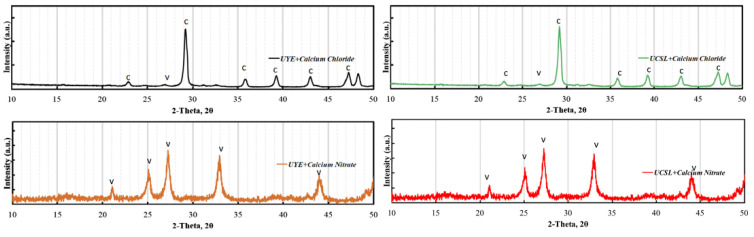
X-ray diffractograms of in vitro biogenic CaCO_3_ precipitates obtained in UYE and UCSL [33], Copyright, 2018, Elsevier, License Number 5763030341971.

Qian et al. [32] used *Bacillus sphaericus* L3 microorganisms to self-repair early cracks in cement-based materials, with the best repair rate occurring with cracks less than 0.4 mm wide. Amiri et al. [33] explored the potential of self-repairing in cement-based materials through biomineralization, primarily microbially induced calcium carbonate precipitation (MICP). They found that the compressive strength of bacterial mortar samples was comparable to pure mortar samples and that using a carbon source (CSL) can promote biomineralization, making biobased admixtures more suitable for specific applications like self-repairing and grouting. Figure 7 shows the X-ray diffraction pattern of in vitro biogenic calcium carbonate induced by *S. pasteurii*, showing that CaCl_2_ precipitated in the UYE medium, resulting in biogenic rhombohedral calcite and globular spherical aragonite. Ca(NO_3_)_2_ in the UYE medium resulted in primarily spherical spherulites, demonstrating the effect of CaCl_2_ on calcium carbonate crystal shape. The study also found that the solubility of the calcium source significantly affects the crystal morphology of *S. pasteurii* induced in vitro biogenic calcium carbonate.

Recently, there has been significant interest in the self-repairing technology for cracks in cement-based materials using microorganisms. Researchers have identified certain microbial species with promising self-repairing capabilities. However, the survival rate of microorganisms directly incorporated into concrete is notably reduced. Therefore, enhancing the survivability of microorganisms in concrete and optimizing their growth conditions have become prominent research focal points. Despite its extensive application prospects, realizing the full potential of this technology requires further efforts in microorganism selection, culture conditions, and other related aspects.

### 2.3. Shape-Memory Alloy

Shape-memory alloy (SMA) technology leverages the martensitic transformation effect to incorporate memory alloys into concrete structures. When cracks form, the strain in the memory alloy increases, allowing a microcomputer to determine the crack width. If the crack width exceeds a predefined limit, the microcomputer can raise the temperature above the martensite reverse transformation temperature, triggering the shape recovery of the SMA. This generates compressive stress at the crack, facilitating self-repair. In cases of unacceptable cracks or excessive loading, the SMA can be heated via electrification, inducing shrinkage and deformation to effectively repair the crack and prevent its propagation [34].

**Figure 8:**
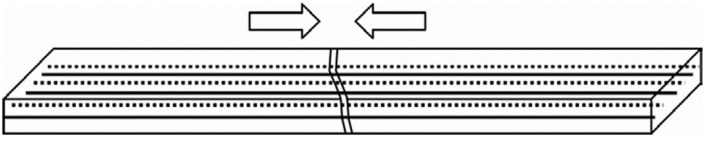
Smart reinforced concrete specimen [34], Copyright, 2006, Elsevier, License Number 5764670701806.

Song et al. [34] introduced the concept of intelligent reinforced concrete using shape-memory alloy wires. These wires are incorporated into martensitic-shaped reinforcement fibers through weaving and tension, as depicted in Figure 8. The resistance of these wires can be monitored to determine stress distribution within the concrete. Applying electricity and heat to the wires can cause multiple wire strands to contract in case of crack formation, mitigating the presence of cracks. This self-repairing mechanism addresses significant cracks, giving concrete structures an intelligence capable of automatic detection and self-repair. Sakai et al. [35] also investigated the self-repairing behavior of concrete blocks using super-elastic shape-memory alloy lines.

**Figure 9:**
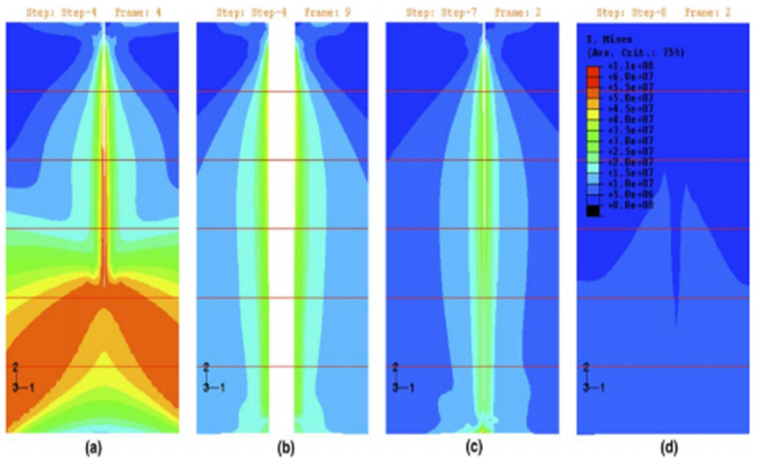
Crack propagation simulation: (**a**) crack halfway through the matrix; (**b**) crack fully opened, matrix halves held together by wires; (**c**) heat causes wires to shorten, which closes crack; (**d**) crack fully closed, and plasticity reversed. SMA wire locations are shown in red [36], Copyright, 2006, Elsevier, License Number 5764671345835.

Burton et al. [36] explored the behavior of self-repairing shape-memory alloy composites through finite-element simulations. Shape-memory alloys (SMAs) are ideal for composites because their properties vary with stress, temperature, and loading history. Their pseudo-elasticity prevents fracturing, and their shape-memory effect allows shape control of the composite when heated. The simulations showed the response of the composites in two scenarios: no cracking and cracking with subsequent healing. The results revealed that pre-strained SMA filaments generate sufficient contraction forces to close heated cracks, even if some martensite is present. Figure 9 illustrates four stages of crack extension and healing: the crack passing halfway through the matrix; the fully open crack held together by the SMA filament; heating causing the SMA filament to shorten and close the crack; and complete crack closure with reversed plastic deformation. This visualizes the role of SMA filament in the self-repairing process.

SMA technology has garnered attention for its role in the remediation of cement-based materials, particularly through real-time crack monitoring enabled by intelligent self-repairing concrete systems. This approach involves raising temperatures to activate the shape-memory effect of SMA and incorporating SMA into strain-hardening cementitious composites (SHCCs). These materials exhibit distinct shape-memory effects and super elasticity, which are beneficial for self-repairing cracks in concrete. However, further advancements are needed in the preparation process, cost reduction, and compatibility to facilitate broader applications of this technology.

### 2.4. Osmotic Crystallization

Osmotic crystallization technology uses active admixtures in concrete or as an external coating. Under certain curing conditions, water moves these admixtures into concrete’s micropores and capillaries, catalyzing cement particle hydration to form insoluble crystals that improve concrete’s bonding strength, impermeability, self-repair, and waterproofing. Active substances include anionic catalysts, waterproofing agents, etc. Anionic catalysts help Ca(OH)_2_, cement particles, and SiO_2_ react to make insoluble hydrates like CaSiO_3_·nH_2_O, filling and sealing cracks for self-repair. The reaction stops once cracks are repaired, and moisture is blocked. If cracks reappear, moisture reactivates the catalyst for new repairs.

**Figure 10:**
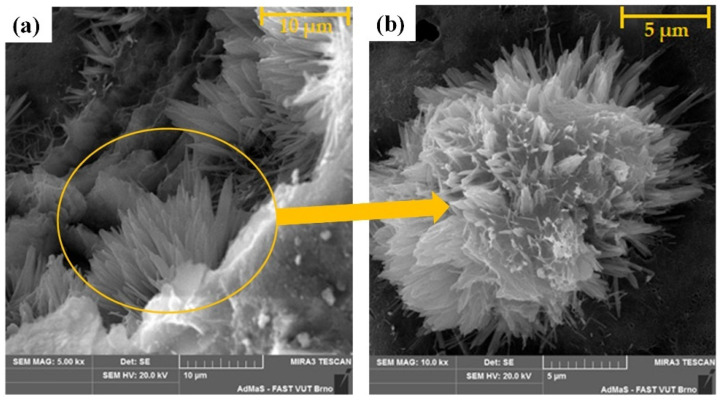
SEM photomicrographs of WL20-CA sample after 540 days curing at 90% relative humidity: (**a**) visible newly formed CA product found in the pore; (**b**) detail of newly formed CA product found in the pore [38].

Dvorkin et al. [37] researched the hydration characteristics and structure formation of cement pastes incorporating metakaolin (MK). They found that SP-MK, doped with superplasticizer (SP), exhibited enhanced reinforcement during the crystallization stage. Similarly, Hodul et al. [38] investigated the impact of crystal admixtures on the properties and microstructure of mortars with by-products. Such admixtures can enhance durability, especially in harsh environments, by reacting with calcium hydroxide to produce mainly calcium carbonate. This can close cracks up to 400 μm within 28 days, reducing water and chemical penetration and improving durability. Combining crystalline admixtures with fly ash further boosts strength and durability by increasing the C-S-H phase content. Figure 10 illustrates new needle-like crystalline admixture products in the pores after 540 days of curing at 90% relative humidity, clustered in a “rose” geometry. These pore-clogging crystals reduce total porosity, disconnect the interconnected matrix pore network, and lower permeability, preventing chloride ion penetration and improving mortar performance when exposed to chlorides.

Osmotic crystallization in self-repairing cement-based materials repairs concrete by forming new crystals at cracks through chemical reactions. This process utilizes unreacted hydration products that react with carbon dioxide to produce mineral crystals, which plug and seal the cracks. Although effective, this method is relatively slow and influenced by environmental conditions. The initial incorporation of specific additives may also increase costs, and improper handling could lead to negative environmental impacts. Future research should focus on developing more effective additives, optimizing formulations, integrating osmotic crystallization with other self-repairing technologies, and advancing intelligent monitoring systems.

### 2.5. Hollow Fiber Technology

The hollow fiber self-repairing method establishes a biomimetic self-repairing network by encapsulating adhesives within glass fiber tubes embedded in a cement matrix. When these tubes fracture, the encapsulated chemicals penetrate the cracks in the matrix, facilitating resealing and repair of the cement. Various adhesives are employed for concrete crack repair, including organic silicon, chloroprene rubber, acrylic ester adhesives, epoxy resin, polyurethane, acrylic, and cement-based materials. Hollow glass fibers offer several advantages, such as ease of preparation, mass production, and low cost. They also demonstrate compatibility with concrete performance and similar linear expansion coefficients, which ensures robust bonding despite temperature fluctuations. The expansion coefficient can be calculated based on the empirical coefficients of their chemical composition, as illustrated in Equation (1).

(1) $$\alpha_{g}=p_{1}\alpha_{1}+p_{2}\alpha_{2}+p_{3}\alpha_{3}\ldots \ldots$$

In the formula, $p_{i}$—percentage of oxides in hollow fiber tubes (%).

$\alpha_{i}$—empirical expansion coefficient of oxides that make up hollow glass fiber.

(1) The hollow glass fiber tube comprises many oxides with stable chemical properties and strong acid and alkali resistance to store and repair the adhesive for a long time.

(2) The strength of hollow glass fiber can also be calculated by the addition formula shown in Equation (2).

(2) $$t=p_{1}t_{1}+p_{2}t_{2}+p_{3}t_{3}\ldots \ldots$$

In the formula, $p_{i}$—percentage of oxides in hollow fiber tubes (%).

$t_{i}$—empirical coefficient of each component.

**Figure 11:**
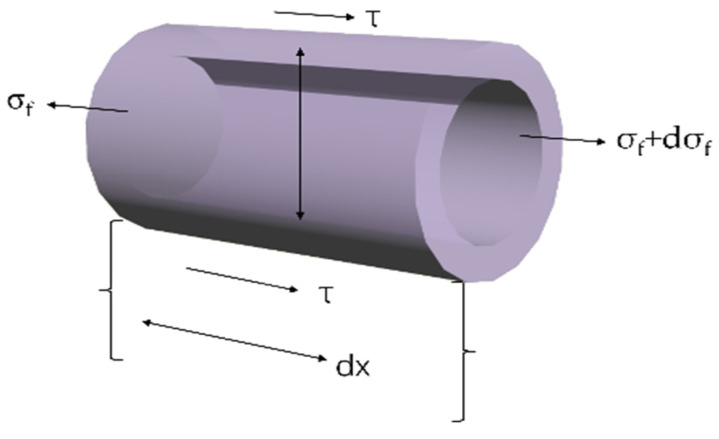
Cooperative working mechanism of hollow glass fiber and concrete.

The cooperative working mechanism of hollow glass fiber and concrete can be described by using the repair fiber shown in Figure 11 as the object to analyze its stress and differential element and establish an equilibrium equation to describe it.

The stress relationship between the bond stress τ and the length of *dx* repair fiber can be obtained from the diagram, as shown in Equation (3):

(3) $$\left[\pi r^{2}-\pi \left(r-t^{2}\right)\right]\times \sigma_{f}+\left(2\pi r\cdot dx\right)\times \tau =\left[\pi r^{2}-\pi \right]\times \left(\sigma_{f}+d\sigma_{f}\right)$$

Equation (3) can be simplified to Equation (4):

(4) $$\frac{d\sigma_{f}}{dx}=\frac{\tau }{t}$$

$\sigma_{f}$—axial stress of repair fiber (MPa).

*τ*—interfacial shear stress between repair fiber and matrix (MPa).

r—repair fiber radius (mm).

t—wall thickness of repair fiber (mm).

It can be seen from Equation (4) that the growth rate of fiber stress along the *X*-axis is proportional to the shear pressure on the interface.

**Figure 12:**
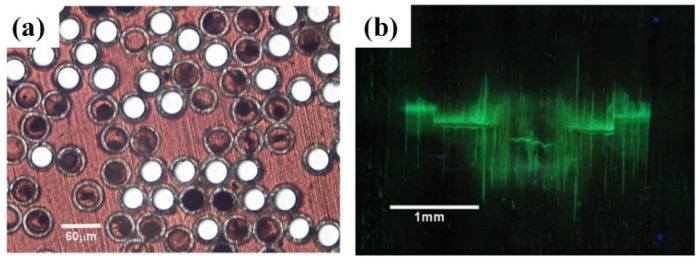
Monitoring of self-repair process by hollow glass fiber technique [40]. Optical micrographs of fibers and composites manufactured at Bristol; (**a**) hollow glass fibers of 60 µm external diameter with a hollowness of 50%; (**b**) comparison of Ultra-Violet Mapping Technique (UMVT) viewed from the front, Copyright, 2005, Elsevier, License Number 5764690053229.

Victor C. Li et al. [39] elucidated the self-repair mechanism of passive smart glass fiber tube cement-based self-repair composites, wherein crack repair is facilitated by releasing the necessary chemicals for crack mending through a curing reaction with air. Pang et al. [40] examined a hollow fiber-reinforced polymer composite with self-repair and damage visibility enhancement capabilities. The study experimentally and analytically investigated the self-repairing behavior of material and damage visualization effects post impact. The repair mechanism relied on impact energy reaching a threshold to fracture hollow fibers, releasing resin for repair. Damage was visualized using ultraviolet fluorescent dyes in the hollow fibers, which exuded and marked the damaged area. Ultrasonic C-scan inspection techniques for rapid damage detection aided this. Mechanical property testing showed that the self-repair process significantly restored bending strength, but repair effectiveness decreased over time. Figure 12a shows a view of the impact surface using UV fluorescent dye, clearly marking the damage area. Figure 12b also shows a view of the impact surface using UVMT, demonstrating a good correlation with ultrasound C-scan for quick damage localization. Overall, the study revealed the effectiveness of UV fluorescent dyes in improving damage visibility in composites, providing a practical means of rapidly detecting and assessing damage.

The advancement of hollow fiber technology in concrete crack repair marks a significant progress in the field of construction materials and structural rehabilitation. This technology provides key benefits, including improved efficiency, durability, adaptability, and environmental sustainability. Future advancements are expected to emphasize the use of novel materials, enhancement of self-repair capabilities, refinement of construction techniques, and the incorporation of multifunctional properties. These developments are poised to offer more reliable and sustainable solutions for the maintenance and restoration of concrete structures.

### 2.6. Microcapsule Self-Repairing Technology

**Figure 13:**
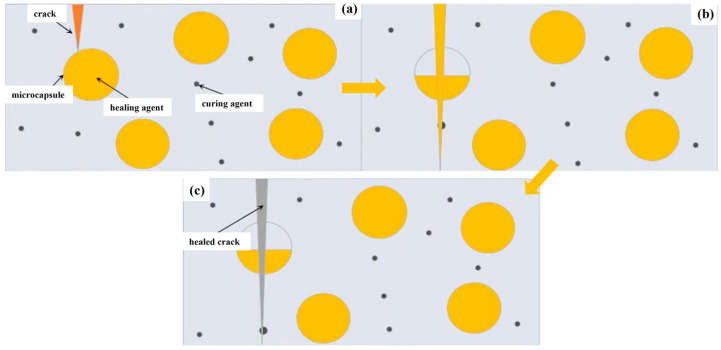
The concept of spontaneous healing. The microencapsulated repair agent is embedded in a structural composite matrix containing a catalyst capable of polymerizing the repair agent. (**a**) Cracks are formed where the damage occurs in the matrix; (**b**) the crack breaks the microcapsule and releases the healing agent into the crack plane through capillarity; (**c**) the healing agent touches the catalyst to initiate polymerization and close the crack surface.

Inspired by the regenerative processes observed in bone tissue healing, S.R. White et al. [41] investigated microcapsule technology to develop an autonomous repair mechanism analogous to the repair of human bones. Their innovative approach involved dispersing microcapsules containing a repair solution and catalyst within a polymer matrix. When cracks occurred, their expansion caused the microcapsules to rupture, allowing the repair fluid to flow into the cracks via capillary action. The repair solution, upon interacting with the embedded catalyst in the polymer, triggered a polymerization reaction, which effectively bonded the fractured surfaces and achieved the desired repair outcome (Figure 13).

**Figure 14:**
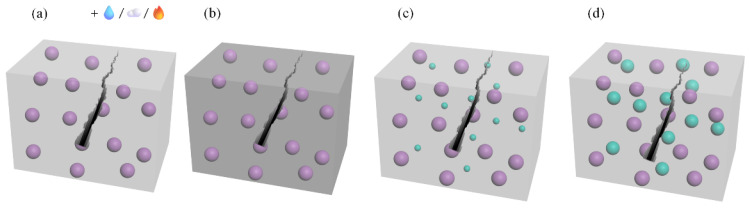
Physical self-repairing mechanism of microcapsules. (**a**) microencapsulated repair agent needs external stimulation to be released; (**b**) the repair agent interacts with the cement-based material internally to be released; (**c**) the repair agent is in contact with the curing agent to repair the cracks; (**d**) different types of microcapsules release different repair agents to repair the cracks together.

Microcapsules in cement-based materials rupture under external loads, releasing a self-repairing agent to enhance the matrix. White’s research examined damaged microcapsules. Standard self-repair methods are shown in Figure 14. In Figure 14a, the repairing agent requires external activation. Commonly used capsule core materials like cyanoacrylate improve cracks when exposed to water or air [34]. In Figure 14b, the repairing agent interacts with concrete to mend cracks, with sodium silicate solution being a joint-improving agent. Upon rupture, the solution reacts with Ca(OH)_2_ in the crack, forming a hydrated calcium silicate product and sealing the crack. In Figure 14c, the matrix is pre-mixed with the curing agent. Upon rupture, the repairing agent engages with the curing agent to mend the crack, with cyclopentadiene dimer being a joint-repairing agent. In Figure 14d, multiple types of microcapsules are added to the matrix. Upon fracture, they rupture, releasing different repair agents that react to achieve matrix repair. Microcapsules are not single objects but a process of encapsulating solid particles, droplets, or gases in an inert shell, shielding them from undesired reactions in the external environment [41].

**Figure 15:**
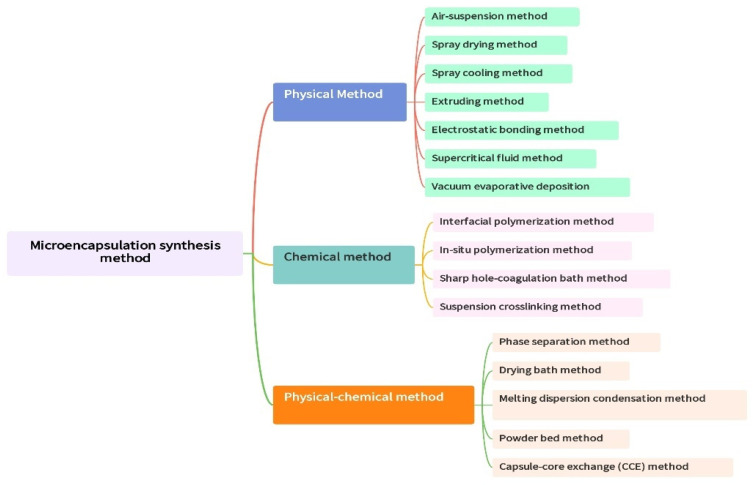
Synthesis of microcapsules.

Various preparation methods exist for microcapsules, resulting in variations in their shapes and sizes. Moreover, different processes can encapsulate multiple core materials. Broadly, the synthesis of microcapsules is categorized into three main methods: physical, chemical, and physicochemical. Figure 15 illustrates some common synthesis approaches.

(1) Spray drying

Spray drying represents a conventional technique for fabricating microcapsules. In this approach, the core and shell materials are dispersed within a solvent to generate a uniformly mixed emulsion. Subsequently, the dispersed liquid is atomized within a stream of hot air at elevated temperatures using an atomization device, resulting in small droplets. Rapid solvent evaporation occurs upon heating, forming a reticular membrane structure comprising the shell material with a sieving effect. Finally, the dried microcapsule powder is obtained through additional drying and curing of the film. The preparation process is primarily delineated into three stages: liquid atomization, product drying, and product separation.

(2) Interfacial polymerization

In 1957, the American DuPont Company synthesized nylon through interfacial polycondensation, marking a significant milestone in polymer chemistry. Since then, interfacial polymerization has been widely adopted for microcapsule fabrication. The method involves dispersing the core and shell material monomers X and Y into a solution using an emulsifier to create an emulsion, either water in oil (W/O) or oil in water (O/W). Subsequently, Y is gradually introduced into the emulsion, with X and Y dissolving in the oil and water phases, respectively. Polymerization occurs at the liquid–liquid interface, forming the capsule shell.

(3) In situ polymerization

The shell material monomer and catalyst are dispersed in the water or oil phases, where the shell material can only be dissolved in one of the phases. It is then mixed with the core material to create an emulsion. During this process, the shell monomers polymerize at the interface of the two phases to form polymers. These polymers do not dissolve in the emulsion but instead precipitate onto the surface of the core material, ultimately creating a complete capsule shell structure that encases the core material to form microcapsules. The primary polymerization methods employed in in situ polymerization include homopolymerization, polycondensation, and copolymerization, among others.

(4) Melting dispersion condensation method

The melting dispersion condensation method involves heating the shell material to a molten state, introducing the core material and stirring it for homogeneity, dispersing the emulsion droplets into a cooling medium, and collecting the microcapsules for further processing. This method produces microcapsules with a uniform particle size and allows for the encapsulation of heat-sensitive, oxidizing, or hydrolytic substances. The thickness and shape of the microcapsule shells can be controlled, regulating their release behavior and physical properties. The method is simple, efficient, and suitable for large-scale production.

Table 1 provides a concise overview of the preparation methods, capsule materials, healing agents, and reported findings in the realm of self-healing concrete. It encapsulates the essence of our study’s innovative approach by detailing the materials and methods that drive the self-healing mechanism, offering a snapshot of the current state of research and contributions to this field.

**Figure 16:**
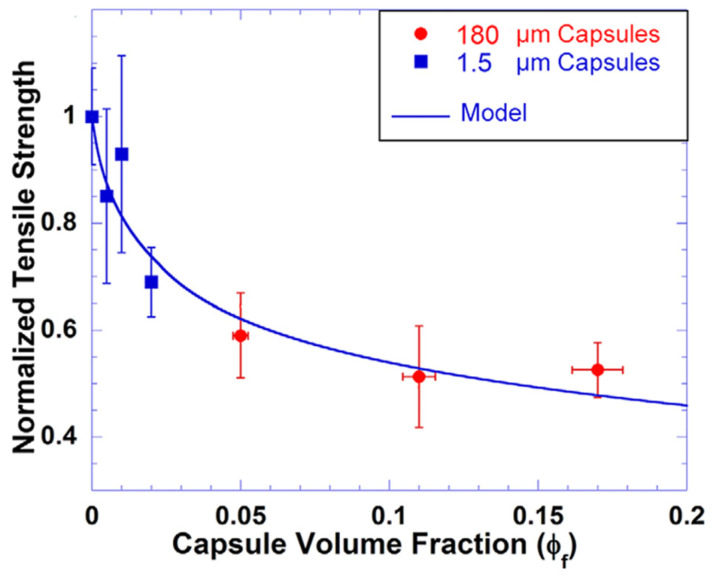
The tensile strength of epoxy/capsule composite for large-diameter capsules (●) and smaller-diameter capsules (■) is compared to the best fit Sudduth model [49], Copyright, 2008, Elsevier, License Number 5764720076987.

The use of microencapsulated self-repair technology enables the self-repair of cement-based materials through different preparation processes. This field of study and application shows great potential for the future. Blaiszik et al. [49] studied nano-capsules in self-repairing materials, focusing on preparation, thermal stability, filling content, elastic modulus, and tensile strength. Optimized treated capsules showed a weight loss below 5% by 100 °C and a significant loss between 150–220 °C, corresponding to the boiling point of DCPD, showing stability at this temperature. Filling content analysis found 78.4% by mass and 94% by volume for DCPD. Smaller capsule sizes had a lesser effect on the elastic modulus of the material. Figure 16 illustrates the impact of different capsule diameters on the tensile strength of epoxy/capsule composites and compares it with the Sudduth model. The results show that tensile strength decreases as capsule diameter decreases, consistent with the Sudduth model. This suggests that the existing model can predict and explain the effect of capsule size on tensile strength despite the size reduction.

**Figure 17:**
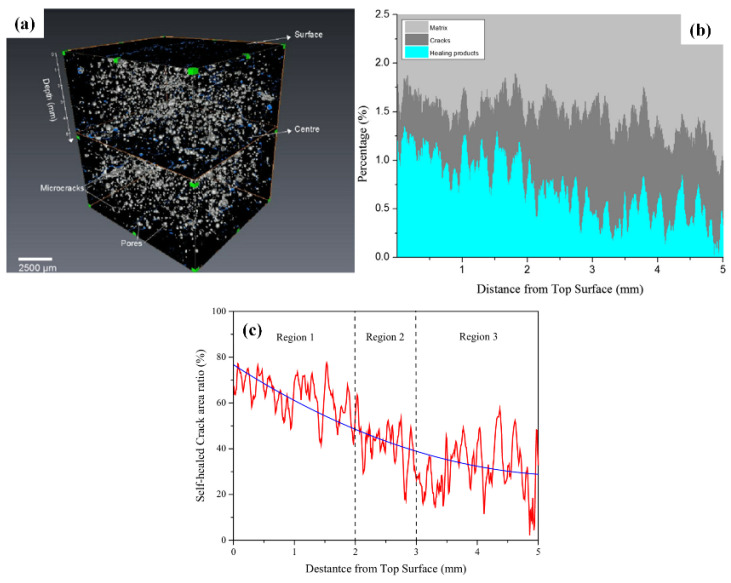
Effect of the crack region on the self-repairing extent. (**a**) Schematic illustration; (**b**) crack area vs. distance from the top surface after 42 days healing; (**c**) self-healed crack area ratio after 42 days healing [50], Copyright, 2008, Elsevier, License Number 5764720384989.

Fang et al. [50] utilized X-ray μCT technology to visually track the self-repair of cracks in cement/microcapsule systems. Traditional crack assessment methods such as microscopy and strength recovery measurement cannot monitor the evolution of healing-related 3D microstructure or quantitatively analyze crack healing. X-ray μCT can provide realistic images of the internal structure of the material and quantitatively analyze crack healing. CSA-based microcapsules were successfully synthesized and used for crack self-repair in cement-based materials, showing excellent surface texture, suitable size, and compatibility with cement paste. The experimental results showed cracks significantly healed with increasing healing time, and the degree and rate of healing depended on the distance from the surface, with rapid and robust self-repair near the surface accompanied by crystal precipitation. After curing, the healing system exhibited significant properties, with a maximum healing rate of 82.60%. Small cracks healed faster than large cracks under the same conditions. The results confirm that the adopted self-repairing system is suitable for cement-based materials. Figure 17 illustrates the impact of crack depth on self-repair. The figure has three fluctuating regions: from the surface to 2 mm (region 1), 40–80% of the crack area was healed; from 2–3 mm (region 2), 20–50% was healed; and in the deepest region (region 3), 5–50% was healed. This indicates that shallower cracks healed more than deeper ones, suggesting the degree and rate of self-repair depends on the distance from the surface, with regions nearer the surface favoring fast and robust healing. This may be due to the cracked area near the surface absorbing more water, enabling a sufficient reaction of the healing agent.

**Figure 18:**
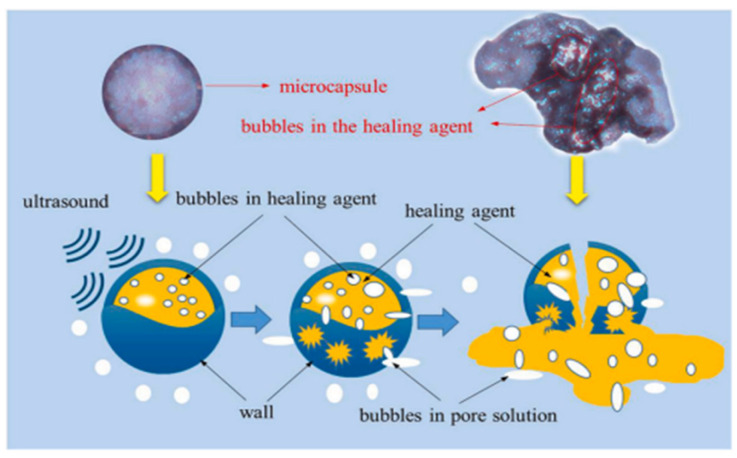
Rupture mechanism of microcapsule triggered by ultrasound [51], Copyright, 2021, Elsevier, License Number 5764720976242.

**Figure 19:**
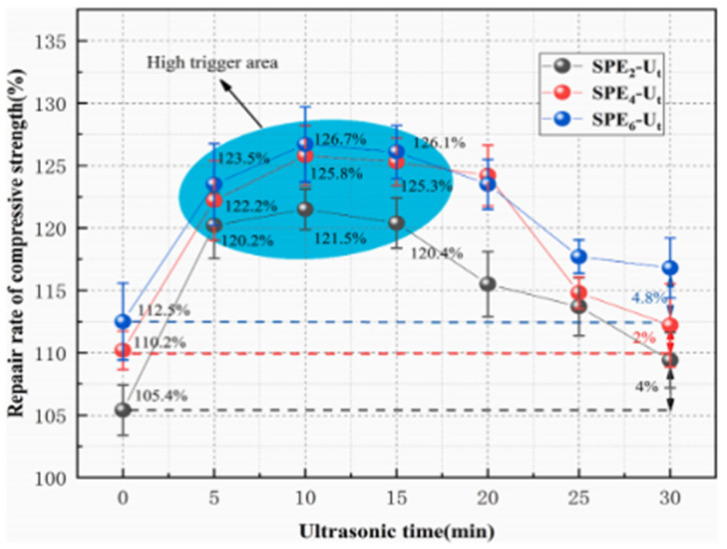
Repair rate of compressive strength of mortars containing different dosages of microcapsules [51], Copyright, 2021, Elsevier, License Number 5764720976242.

Xu et al. [51] studied ultrasound as a trigger for self-repairing cement-based materials with microcapsules. Ultrasonic waves effectively improved the triggering and release efficiency of UF/E microcapsules, with most microcapsules partially fragmenting and releasing encapsulated restorative. In contrast, mechanical triggering had significantly lower efficiency. Ultrasonic triggering also improved the strength-repair rate of self-repairing mortar, with a strength enhancement 2–4 times higher than mechanically triggered specimens. Ultrasound positively and negatively affected the pore structure of microcapsule-doped self-repair mortar, filling harmful pores with repair agents and slightly damaging the cement matrix microstructure. There is a theoretical optimal trigger time for ultrasonic triggering due to its dual effect on the pore structure. Under the conditions of this study (40 kHz, 0.2 w/cm^2^), the optimal treatment time was about 10 min. However, further research is needed to understand the effects of ultrasonic frequency and power on the repair effect of microcapsule-doped self-repairing cement-based materials. Figure 18 illustrates the pore size distribution and cumulative pore volume of mortar containing 6% microcapsules at the optimum ultrasonic treatment time. The results show that the volume of less deleterious pores (20–50 nm), deleterious pores (50–200 nm), and very harmful pores (200–500,000 nm) of the ultrasonically treated SPE_6_-U_10_ decreased, especially in the range of 10,000–3500,000 nm, compared to the non-ultrasonicated SPE_6_-M. This is due to the increased release rate of microcapsules from the ultrasonically-triggered microcapsule-doped self-repairing mortar, which fills and repairs more macropores. Figure 19 illustrates the compressive strength-repair rate of pre-damaged specimens with varying microcapsule amounts at different ultrasonic triggering times. The strength-repair rate increased with higher microcapsule dosage, but the enhancement was slowed. For instance, the rates of SPE_2_-U_10_, SPE_4_-U_10_, and SPE_6_-U_10_ were 25.3%, 29.6%, and 30.5% higher than SPE_0_-U_10,_ respectively. However, SPE_6_-U_10_ only had a 0.9% advantage over SPE_4_-U_10_. Therefore, from a practical standpoint, excessive microcapsules are unnecessary.

**Figure 20:**
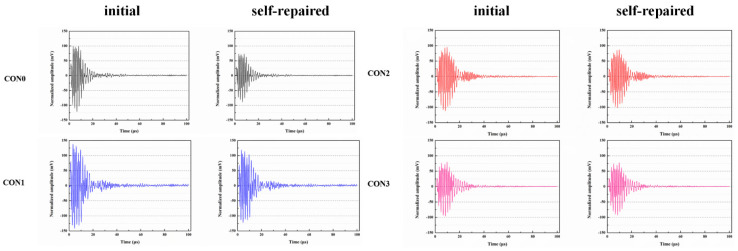
The ultrasonic frequency of the concrete containing microcapsules before and after self-repairing (100 freeze–thaw cycles) [55], Copyright, 2021, Elsevier, License Number 5773961411787.

**Figure 21:**
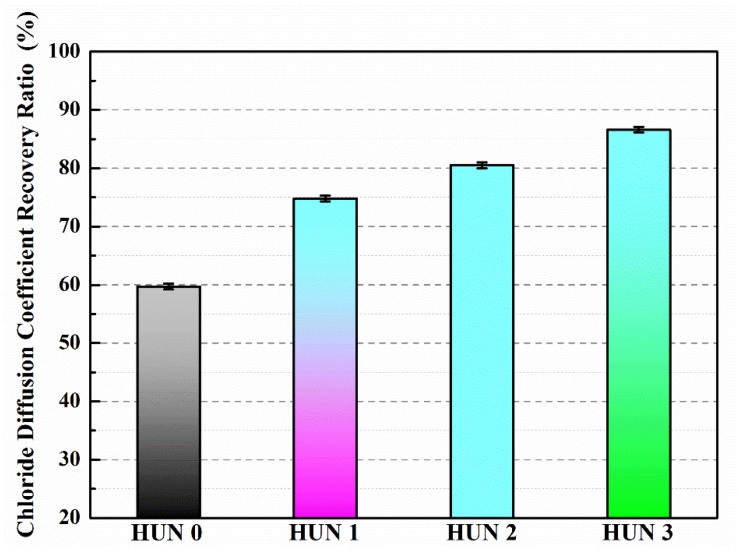
The chloride diffusion coefficient recovery ratio of concrete after 14 d of self-repairing (180 dry–wet cycles) [56], Copyright, 2021, Elsevier, License Number 5774040151380.

Du et al. [47,48,52,53,54,55,56,57,58] initially investigated the impact of preparation temperature, stirring rate, and mass ratio of paraffin and isocyanate (TDI) on TDI microcapsules. The most effective microcapsules were achieved with a paraffin/TDI mass ratio of 1:2, a stirring rate of 600 rpm, and a temperature of 75 °C. During the second phase, the self-repairing capability of paraffin/TDI microcapsule mortar in various external conditions (humidity and temperature) was examined by comparison of the pore size distribution, mechanical properties, impermeability, and surface crack width of regular and microcapsule mortar. The study found that the curing reaction of TDI in the self-repairing process of microencapsulated mortar followed the quasi-secondary kinetic model, showing that humidity and temperature significantly influenced the self-repairing ability of mortar. Introducing nano-SiO_2_/paraffin/PE wax as the shell material to encapsulate TDI enabled the microcapsule to fully self-heal surface cracks in the mortar within 4 h, indicating its effective self-repairing capability for cement-based material cracks. An investigation was conducted on the impact of composite shell microcapsules on the frost resistance and self-repairing capabilities of concrete during freeze–thaw cycles. Figure 20 illustrates the ultrasonic frequencies of concrete with microcapsules before and after self-repair. The peak amplitudes of the primary frequency for CON0 (control concrete), CON1 (added 8.4 kg/m^3^ of TM1), CON2 (added 8.4 kg/m^3^ of TM2), and CON3 (added 8.4 kg/m^3^ of TM3) were 10.17 mV, 12.90 mV, 9.22 mV, and 8.69 mV, respectively. Concrete has internal pores, which can be filled by adding an appropriate amount of TM1 (paraffin-encapsulated TDI microcapsule). This process optimizes the concrete gradation and enhances the maximum amplitude of the dominant frequency. If the particle size of TM2 (paraffin/PE wax-encapsulated TDI microcapsule) or TM3 (nano-SiO_2_/paraffin/PE wax-encapsulated TDI microcapsule) added is more significant than that of TM1, it will decrease the concrete compactness and diminish the maximum amplitude of the primary frequency. The study demonstrated that reducing the pore size and raising the density of the microcapsules enhanced the ability of concrete to withstand freeze–thaw cycles. The composite shell TDI microcapsules improved the frost resistance and self-repairing capacity of concrete, even after undergoing 100 freeze–thaw cycles. Microcapsules were created in the third phase by utilizing IPDI as the core material and paraffin wax, PE wax, and silica nanoparticles as the shell material to enhance the resistance of concrete to sulfate attack and self-repairing capabilities. The study analyzed the effects of sulfate dry and wet cycling on concrete by examining mass loss, mechanical properties, impermeability, and pore size distribution. The recovery rates of HUN0 (control concrete), HUN1 (added 8.4 kg/m^3^ of MS1), HUN2 (added 8.4 kg/m^3^ of MS2), and HUN3 (added 8.4 kg/m^3^ of MS3) were found to be 59.7%, 74.8%, 80.5%, and 86.6%, respectively (as shown in Figure 21). The microcapsule concrete showed a significantly higher recovery rate than HUN0, indicating superior self-repairing properties. The chloride diffusion coefficient recovery of HUN3 was higher than that of HUN1 and HUN2 due to the superior core content, densification, and micromechanical characteristics of MS3 (paraffin/PE/nano-SiO_2_-encapsulated IPDI microcapsule) compared to MS1 (paraffin-encapsulated IPDI microcapsule) and MS2 (paraffin/PE wax-encapsulated IPDI microcapsule). The study found that concrete containing nano-SiO_2_/paraffin/PE wax-encapsulated IPDI microcapsules demonstrated strong sulfate attack resistance and self-repairing properties. The melt condensation method prepared nano-CaCO_3_/ceresine wax containing E-44 epoxy resin microcapsules (WM2). The results revealed that WM2 exhibited effective self-repairing capabilities for cracks on the mortar surface. Cracks with an initial width of less than 0.35 mm on the mortar surface were completely self-repaired within three days.

**Figure 22:**
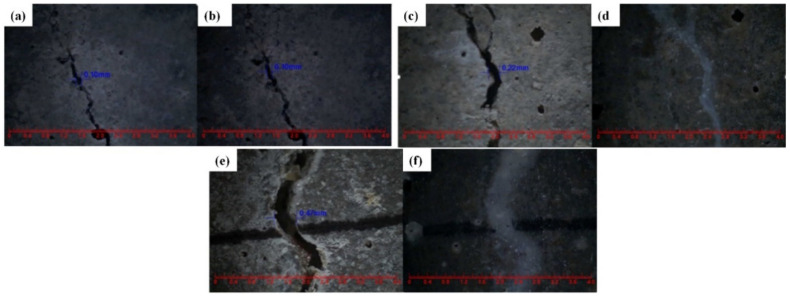
Surface cracks width of mortars. (**a**) SJ-0, (**b**) SJ-0 for 15-day self-repair, (**c**) SJ-1, (**d**) SJ-1 for 15-day self-repair, (**e**) SJ-2, and (**f**) SJ-2 for 15-day self-repair [47].

**Figure 23:**
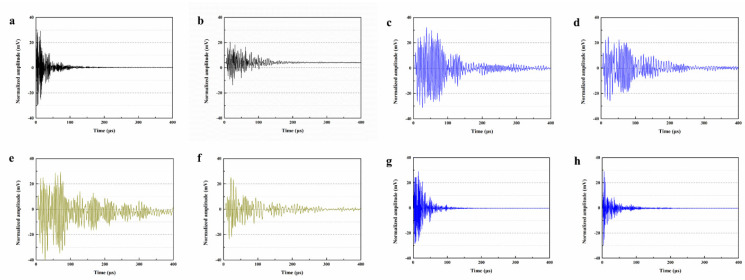
Ultrasonic waveform of S0–S3. (**a**) The initial ultrasonic waveform of S0, (**b**) the ultrasonic waveform of self-healed S0, (**c**) the initial ultrasonic waveform of S1, (**d**) the ultrasonic waveform of self-healed S1, (**e**) the initial ultrasonic waveform of S2, (**f**) the ultrasonic waveform of self-healed S2, (**g**) the initial ultrasonic waveform of S3, and (**h**) the ultrasonic waveform of self-healed S3 [48].

Microcapsules have repair agents that can automatically repair cracks. However, the tremendous mechanical strength of thermoset polymer shells makes it challenging for the microcapsules to break open when needed. Previous research involved creating microcapsules by enclosing epoxy resin with microcrystalline wax, enhancing the mechanical characteristics of the microcapsules, but a rupture hazard remained. The study attempted to improve the durability of microcapsules by including nanomaterials in the synthesis of shell materials. However, the microcapsules did not consistently appear at the crack tip. The authors suggested incorporating magnetic nanoparticles into the shell material to create microcapsules that can be triggered by electromagnetic fields, resulting in the shell melting and breaking owing to elevated temperature. Novel electromagnetic-induced rupture microcapsules (DWMs, mixed with 3% of cement mass) were created using Fe_3_O_4_ nanoparticles and PE wax as the shell material and epoxy resin as the core material. The study demonstrated that adding these microcapsules enhanced the self-repairing capability of mortar (as shown in Figure 22). To decrease energy consumption and CO_2_ emissions in cement preparation, limestone, calcined clay, and gypsum were used as SCMs to replace 50% of ordinary Portland cement (OPC). LC3 mortar was then created, and microcapsules were added, making up 4% of the total mass of cement-based materials. The study demonstrated that LC3 mortar with microcapsules had superior self-repairing capabilities compared to OPC mortar with microcapsules under identical settings. The self-repairing capacity of microcapsules in LC3 mortar increased with increasing temperature. Following this, three different microcapsules (M1, M2, and M3) were created using IPDI as the core material and PE wax, PE wax/nano-CaCO_3_, and PE wax/iron powder as the shell material. Microcapsules were incorporated into LC3 mortar to evaluate their impact on the mechanical characteristics, pore structure, and permeability of the mortar. Figure 23 demonstrates the ultrasonic waveforms of the S0–S3 samples, including the initial waveform and the waveform after self-repair (S0: the control specimen, S1: mixed with 4% M1, S2: mixed with 4% M2, and S3: mixed with 4% M3). From the figure, it is evident that (1) the S0 samples showed no signs of crack healing after self-repair, indicating that the cement-based materials alone cannot heal the surface cracks; (2) S1, S2, and S3 samples showed complete healing of the cracks after self-repair, indicating that the microcapsules can self-heal surface cracks; and (3) the M3 microcapsules exhibited the largest crack healing width after self-repair, showing the best self-repairing effect. In summary, the results of the data analysis in Figure 23 support the effectiveness of microcapsules in improving the self-repairing ability of cement-based materials, especially the significant effect of microcapsule M3 containing iron powder under an applied magnetic field [47,48,52,53,54,55,56,57,58].

**Figure 24:**
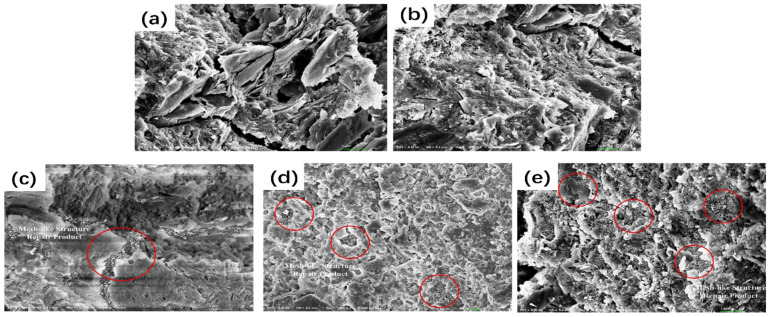
Microstructure of concretes. (**a**) SPE0, (**b**) SPE1, (**c**) SPE2, (**d**) SPE3, and (**e**) SPE4.

Du et al. [59] presented a method for enhancing the frost resistance and self-repair capabilities of LC3 concrete using microcapsules. The microcapsules, with isophorone diisocyanate (IPDI) as the core and shells composed of microcrystalline wax, ceresine wax, and CaCO_3_, were integrated into the concrete matrix. The research demonstrated that the addition of these microcapsules significantly improved the freeze–thaw resistance of LC3 concrete, with the YX3 (shell: microcrystalline wax, ceresine wax, and CaCO_3_; core: IPDI) formulation showing the most promise. Figure 24 reveals the microstructural differences in concrete samples with and without self-repair microcapsules after exposure to 200 freeze–thaw cycles and a subsequent 14-day self-repair period. The SEM images depict the internal structure of control samples (SPE0: OPC concrete without any LC3 components or microcapsules; SPE1: LC3 concrete without microcapsules) with unfilled pores and microcracks, indicating damage from freeze–thaw cycles. In contrast, samples containing microcapsules (SPE2: LC3 concrete containing YX1 (shell: microcrystalline wax; core: IPDI); SPE3: LC3 concrete with YX2 (shell: microcrystalline wax and ceresine wax; core: IPDI); and SPE4: LC3 concrete containing YX3) show a denser microstructure with mesh-like repair products, particularly in SPE4 with YX3 microcapsules, suggesting effective self-repair. After 200 freeze–thaw cycles, the concrete with microcapsules exhibited lower mass loss and higher compressive strength retention compared to control samples. Researchers evaluated the microcapsules’ performance under extreme conditions through mass loss measurements, compressive strength assessments, and ultrasonic waveform analyses. The results indicated that the microcapsules effectively released the IPDI healing agent upon cracking, leading to the repair of microcracks within the concrete. Ultrasonic testing revealed that the concrete with microcapsules had a higher amplitude, implying better compaction and fewer internal defects.

In conclusion, the study highlights the potential of microencapsulated self-repair technology to increase the durability and lifespan of concrete structures, especially in cold regions. This approach not only bolsters resistance to environmental stressors but also provides an autonomous repair mechanism, which could lead to reduced maintenance costs and extended service life of infrastructure. The findings contribute to the advancement of sustainable and smart construction materials capable of withstanding and self-repairing in harsh environments.

Overall, research has demonstrated the potential of employing various microcapsules for concrete damage repair. For example, some microcapsules use silica sol or sodium silicate solution as core materials [60]. Additionally, researchers have explored adjusting the mechanical properties of microcapsules by varying shell materials to suit different environments [61,62]. Technologically, the production of microcapsules has become highly automated and scalable. Microfluidics, for instance, enables precise control over microcapsule size and shell thickness [63], while 3D printing facilitates the creation of microcapsules with intricate structures [64]. These advancements suggest that producing microcapsules tailored to diverse concrete repair needs is feasible. However, challenges and limitations persist. Some microcapsules may compromise concrete strength or alter other properties. Moreover, optimizing the triggering mechanisms of microcapsules is crucial for ensuring their efficient and reliable operation in practical applications. Despite these technological and performance challenges, evidence supports the potential development of microcapsules that can deliver a range of reparative agents for various types of concrete damage. Future research should focus on optimizing microcapsule design, improving efficiency and reliability in real-world scenarios, and expanding their applicability.

### 2.7. Comparison and Analysis of Advantages and Disadvantages of Self-Repairing Technologies

**Figure 25:**
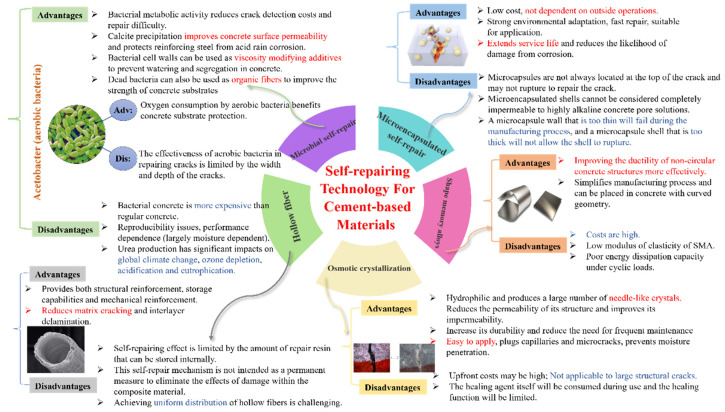
Analysis of advantages and disadvantages of self-repairing technology.

Implementing self-repairing technology in cement-based materials represents an innovative approach to addressing the challenges of cracking and deterioration in concrete. Although these technologies show considerable promise, it is essential to assess their cost-effectiveness, long-term performance, and practical applicability before adopting them in construction projects. Figure 25 illustrates the advantages and disadvantages of the five self-repairing methods discussed in this article.

## 3. Self-Repairing Performance Test Method

Research on the self-repair performance of cement-based materials has gained considerable attention. Achieving self-repair of cracks in these materials and structures requires a variety of methods, which depend on factors such as material type, environmental conditions, and performance requirements. Additionally, other structural materials have distinct performance and self-repair needs, necessitating different repair processes and evaluation techniques. A significant challenge in developing self-repairing materials lies in using conventional testing techniques to assess the effectiveness of various repair processes. This section primarily discusses the testing methods employed to evaluate the self-repairing capabilities of fractures under environmental or mechanical influences.

### 3.1. Characterization of Microencapsulation Properties and Analysis of Influencing Factors

Various methods have been developed to identify microcracks in the cement matrix, including optical microscopy (OM), scanning electron microscopy (SEM), and acoustic emission microscopy (AEM) [65]. However, some of these techniques may cause material damage when detecting minor flaws in the structure [66]. To avoid these potential negative consequences, it is generally advisable to obtain a sample of the subject design. The following section outlines various techniques for identifying cracks in cement-based materials.

1. Optical microscope

An optical microscope (OM) is utilized to identify microcracks in cement materials under natural light. The visibility of these cracks is influenced by factors such as the angle of light, surface characteristics, intensity, type of cement-based material, and the height and shape of the crack [67]. The use of colored resin during the impregnation process can enhance the visibility of microcracks, thereby improving the reliability of the results [68]. Yellow fluorescent epoxy resin increases the detectability of microcracks in cement-based composites. Fluorescent resins, created by combining fluorescent dyes with transparent resins, provide contrast when viewed under UV light in an optical microscope and reinforce fractured materials by filling the cracks [69]. To ensure all cracks are filled, the sample is impregnated under vacuum conditions.

**Figure 26:**
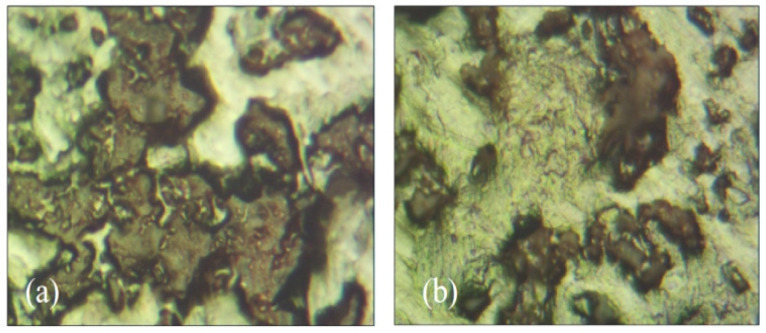
(**a**) Optical microscope image of CM1-0; (**b**) optical microscope image of TM1-20 [70], Copyright, 2018, Elsevier, License Number 5764751258892.

Thiruvenkitam et al. [70] investigated the impact of using foundry sand (UFS) as a partial replacement for fine aggregate on the properties of concrete. The study examined fresh, mechanical, and durability properties along with microstructural analysis. Figure 26a displays an optical microscope image of CM1-0, where UFS is not added. The incorporation of UFS led to a more compact crystal structure, attributed to the higher silica content, which filled and eliminated pores, thereby enhancing the density and properties of the concrete. Figure 26b shows an optical microscope image of TM1-20, where 20% UFS is added. The addition of UFS increased the silicate content, resulting in a more regular crystal structure with a flower-like morphology. This structure helped to fill or eliminate pores, improving the compactness and mechanical properties of the concrete. Furthermore, the formation of CASAH gel was more optimized in TM1-20 compared to the control, contributing to improvements in mechanical and durability properties. However, an excess of UFS reduced the physical strength due to the presence of fines and insoluble residues.

**Figure 27:**
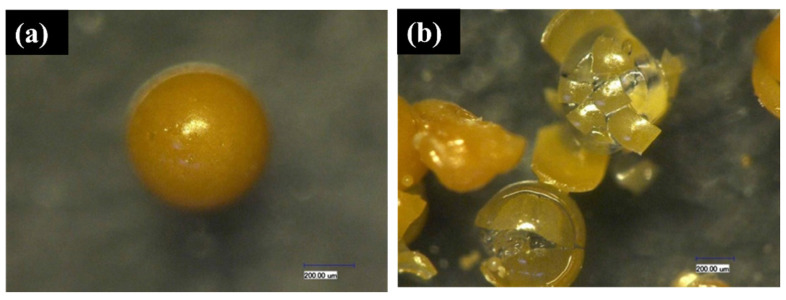
OM images of (**a**) a single microcapsule and (**b**) broken microcapsules compressed by two parallel glass sheets [71], Copyright, 2016, Elsevier, License Number 5764760062680.

**Figure 28:**
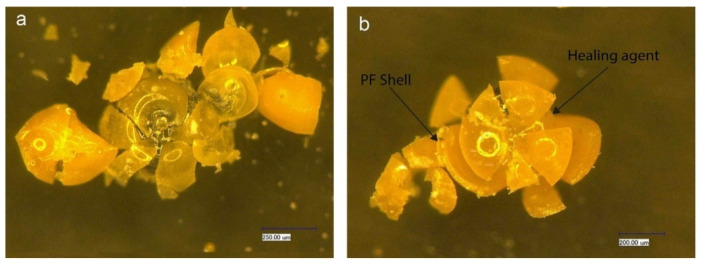
OM images of rupture of PF/DCPD microcapsules after immersion in simulated pore solution for (**a**) 3 h and (**b**) 48 h [71], Copyright, 2016, Elsevier, License Number 5764760062680.

Lv et al. [71] introduced a novel synthesized polymer microcapsule and investigated its feasibility for self-repairing cement materials. Their findings demonstrated that these microcapsules can be activated by cracks, releasing a repair agent to achieve the self-repairing function in cement materials. The researchers also examined the morphology, size distribution, chemical stability, and bonding properties of microcapsules to the cement matrix. Figure 27 illustrates the regular spherical shape and smooth surface of the microcapsules, along with their brittle fracture mode upon rupture—key features for self-repairing applications in cement-based materials. Figure 28 presents OM images of PF/DCPD microcapsules after immersion in a simulated pore solution for 3 and 48 h. As shown in Figure 28a, the microcapsules remained intact with a smooth surface and no apparent rupture after 3 h. In contrast, Figure 28b shows that after 48 h, the microcapsules ruptured, releasing DCPD and forming a noticeable dark area. This indicates that while the microcapsules exhibit stability in the simulated pore solution, they will rupture after prolonged immersion, releasing the repair agent and thus enabling the self-repair function.

2. Scanning electron microscope

The scanning electron microscope (SEM) is a high-resolution technique capable of detecting crack widths as small as 100 nm. In contrast, the optical microscope (OM) has limited resolution and cannot detect very small, unimpregnated cracks. It is essential to thoroughly dry samples before SEM crack identification to prevent subsequent drying shrinkage microcracks [72], which are characterized by sharpness, grinding, and polishing artifacts. Cracks are visible as black lines in SEM images, displaying the shadow of the crack [68]. Similar to the OM approach, SEM allows for the impregnation of samples with resin, enabling samples prepared with OM to be evaluated with SEM [66]. SEM is more effective than OM in detecting intricate details in impregnated samples. Fissures in the filled resin exhibit strong contrast under SEM, especially when using a backscattered electron (BSE) detector [72].

**Figure 29:**
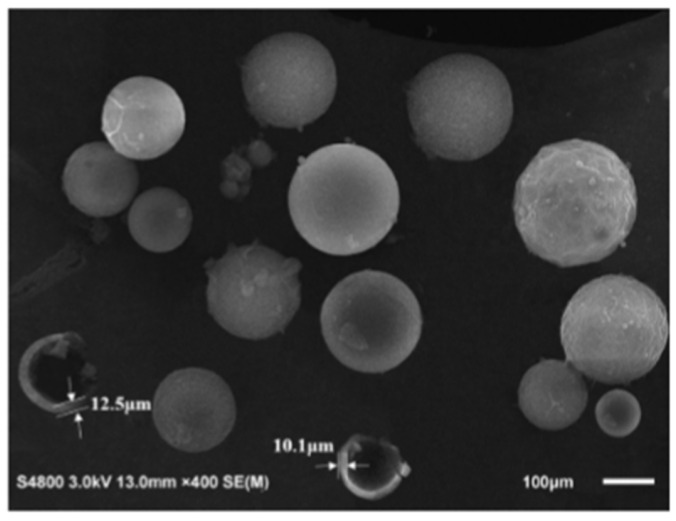
SEM image of the microcapsules [52], Copyright, 2019, Elsevier, License Number 5773971493998.

As observed in Figure 29, the microcapsules exhibit a regular spherical shape with a rough surface, which contributes to a strong bond between the microcapsule shells and the cement matrix. The particle size distribution of the microcapsules ranges from 75 to 150 μm, consistent with the results from the particle size distribution test. The images show that a ruptured microcapsule with a diameter of 100 μm has a shell thickness of 10.1 μm, while a ruptured microcapsule with a diameter of 120 μm has a shell thickness of 12.5 μm. This indicates that the shell thickness of the microcapsules is approximately one-tenth of their diameters. These findings suggest that the microcapsules, prepared under optimal parameters, have a high diameter-to-shell thickness ratio and can provide the desired TDI encapsulation capacity. Overall, Figure 29 illustrates the morphology, particle size distribution, and shell thickness of the microcapsules through SEM images, which are essential for understanding the preparation quality of the microcapsules and their potential performance in concrete self-repairing applications.

**Figure 30:**
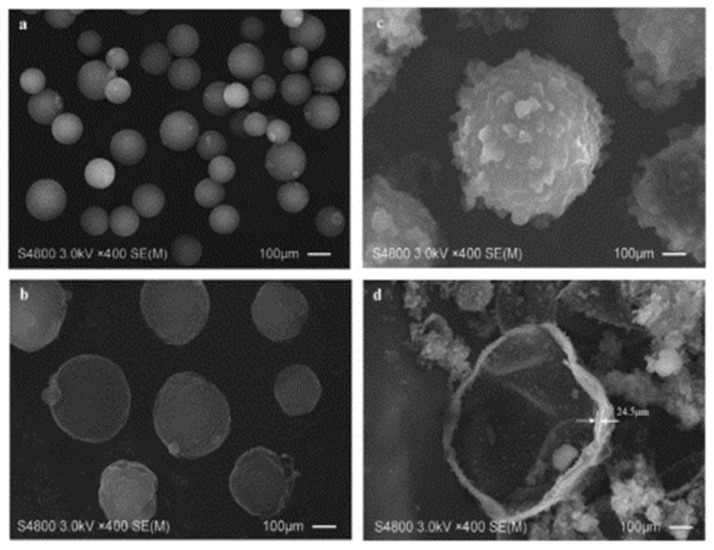
SEM image of microcapsules: (**a**) MC1, (**b**) MC2, (**c**) MC3, and (**d**) fractured MC3 [53], Copyright, 2020, Elsevier, License Number 5774030286674.

Figure 30 illustrates the morphological characteristics of three types of microcapsules—MC1 (paraffin/TDI), MC2 (paraffin/PE wax), and MC3 (nano-SiO_2_/paraffin/PE wax)—along with their fracture surfaces as shown in SEM images. MC1 exhibits a regular spherical shape with a small particle size and a smooth surface. MC2 has a larger particle size and a rough surface, which is mainly due to the low viscosity and good dispersion of paraffin waxes. MC3 displays the largest particle size and a very rough surface, attributed to the addition of nanosized SiO_2_, which increases the mixture viscosity. The fractured image of MC3 further highlights the thickness and roughness of its shell structure. These morphological features are closely related to the preparation materials and process parameters of the microcapsules, influencing their performance and application.

**Figure 31:**
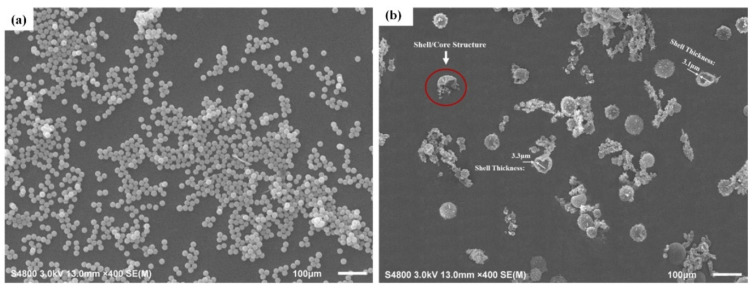
SEM images of microcapsules: (**a**) WM1 and (**b**) WM2 [57].

Figure 31 shows the internal composition and outer appearance of E-44 epoxy resin microcapsules. The surface of WM1 (ceresine wax shell material) in Figure 31a is smooth, with a consistent spherical shape and a particle size of approximately 20 µm. Figure 31b shows that the surface of WM2 is rough and significantly larger, with a particle size distribution around 50 µm. The increased viscosity and reduced dispersion resulting from the ceresine wax and nano-CaCO_3_ mixture led to a rougher and more granular surface for the microcapsules. WM2 exhibits a distinct core–shell structure with a thin outer layer, which encases the E-44 epoxy resin for optimal storage.

3. Fourier Transform Infrared Spectroscopy (FTIR)

FTIR spectroscopy is used to analyze the chemical composition of microcapsules by detecting the light that is scattered or absorbed by the sample at various wavelengths. This technique is valuable for evaluating the stability, sustainability, and effectiveness of microencapsulation in applications related to microencapsulated self-repairing technologies. By identifying absorption peaks at different wavelengths, FTIR testing can determine the chemical composition and concentration of the microencapsulated shell layer, thereby ensuring the reliability and effectiveness of the microcapsules in self-repairing applications.

**Figure 32:**
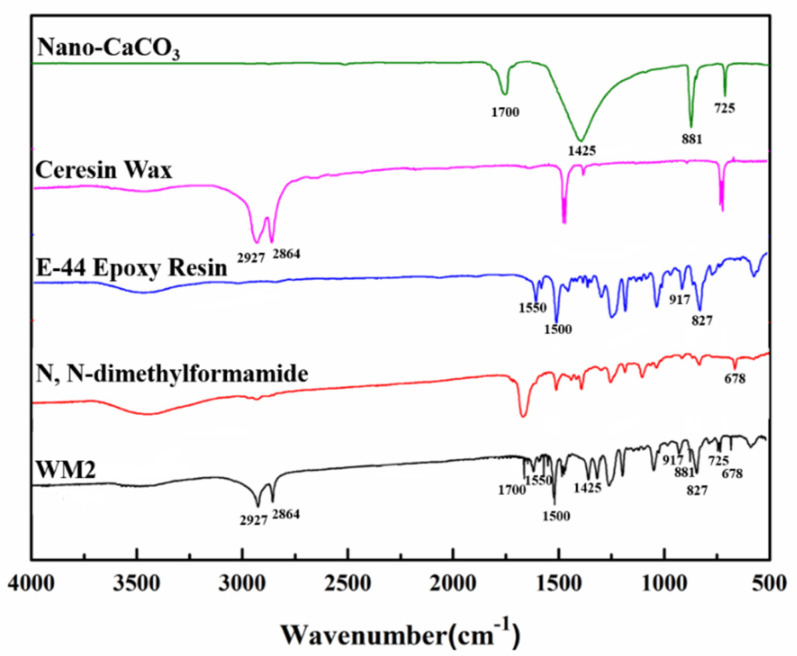
FTIR spectra of nano-CaCO_3_, ceresine wax, E-44 epoxy resin, N, N-dimethylformamide, and WM2 [57].

Figure 32 presents the FTIR spectra for ceresine wax, N,N-dimethylformamide, WM2, nano-CaCO_3_, and E-44 epoxy resin. The peak at 1700 cm^−1^ corresponds to the stretching vibration of the C=O bond in nano-CaCO_3_, while the peak at 1425 cm^−1^ indicates the antisymmetric stretching vibration of the C–O bond in CO_3_^2−^. The amide I band peak reveals the presence of a small amount of N,N-dimethylformamide encapsulated in WM2. The combined SEM and FTIR results confirm that E-44 epoxy resin has been successfully encapsulated within the nano-CaCO_3_/ceresine wax composite shell.

FTIR testing is a crucial tool in the self-healing technology of microcapsules made from cementitious materials. It helps identify and confirm the composition of the core and shell components in microcapsules, ensuring they align with desired specifications. For example, infrared spectroscopy confirmed that the central part of the microcapsule was made of epoxy resin, while the outer layer was made of urea-formaldehyde resin. It also provides data on the molecular structure of self-healing agent molecules, helping to elucidate the interaction mechanism between the agent and cementitious materials. It can also be used to monitor chemical reactions during the self-healing process, providing an experimental foundation for controlling the self-healing process. Quality control is also achieved through infrared spectroscopy, ensuring the uniformity of composition and structure, thereby ensuring the stability and dependability of the self-repair technology. Overall, infrared spectroscopy is a vital tool in the self-healing technology of microcapsules made from cementitious materials.

4. Raman spectra

Raman spectroscopy is based on the inelastic scattering of monochromatic light, typically provided by a laser. Inelastic scattering occurs when photons in the monochromatic light interact with the sample, resulting in a change in their frequency. The sample absorbs the laser photon and then re-emits a photon with a frequency different from that of the original light, a phenomenon known as the Raman effect. This shift allows for the study of molecular vibrations, rotations, and other low-frequency transitions. Raman spectroscopy is versatile and can analyze samples in solid, liquid, and gaseous states.

**Figure 33:**
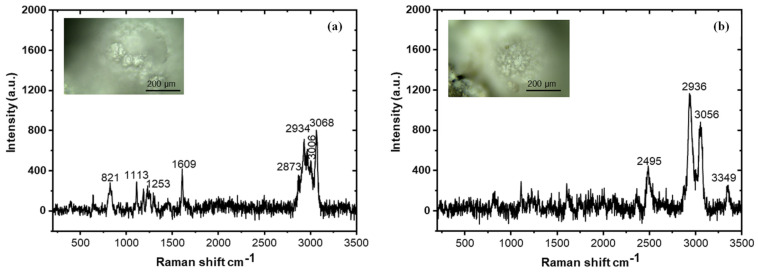
(**a**) Raman spectrum of epoxy of a broken microcapsule in cement [73], Copyright, 2023, Elsevier, License Number 5764870108814. (**b**) Raman spectrum of an unbroken microcapsule in cement [73], Copyright, 2023, Elsevier, License Number 5764870294117.

Mi et al. [73] utilized Raman spectroscopy to investigate the repair behavior of microcapsules in self-repairing cement-based systems. Their study demonstrated that Raman spectroscopy can effectively differentiate between unruptured and ruptured microcapsules, enabling the monitoring of chemical composition changes during the self-repair process. The research also evaluated the titanium dioxide content in the microcapsules by analyzing the intensity changes in Raman peaks, which correspond to the degree of rupture. Figure 33a presents the Raman spectrum of a ruptured epoxy resin microcapsule embedded in cement, revealing four prominent peaks that indicate epoxy resin as the primary core component. Figure 33b displays the Raman spectrum of intact microcapsules in cement, highlighting urea-formaldehyde (UF) as the main chemical component. The absence of peaks associated with the microcapsule core material (e.g., epoxy resin) in this spectrum suggests that the thickness of the shell material was sufficient to prevent the laser from detecting scattered light from the core.

### 3.2. Restoration of Macroscopic Characteristics of Cement-Based Materials Using Microcapsules

1. Mechanical properties

Mechanical testing involves fracturing prismatic specimens and evaluating their mechanical properties both before and after repair at various ages. The standard testing procedure is based on fracture behavior theory and uses crack mouth opening displacement (CMOD) to control crack propagation and assess the self-repairing capability of materials under different crack conditions. To evaluate the mechanical properties of repaired cracks, the three-point bending test is performed again after the specimen has undergone a repair period. Comparing the test results of unrepaired cracks with those of repaired cracks is crucial for assessing the recovery of mechanical properties. Additionally, both the repaired and original specimens should be subjected to the same repair conditions and ages. Another three-point bending test is then conducted, where the specimen is initially loaded to a specific deformation displacement, unloaded, and reloaded. The highest load achieved during the second loading is recorded as the maximum bending stress for the specimen with unrepaired cracks.

Typically, incorporating microcapsules into cement-based materials leads to a decrease in their compressive strength. Moreover, the degree of this decrease is directly correlated with the quantity of microcapsules that are included. Dong et al. [74] investigated the compressive strength of cement-based materials that were microencapsulated. They conducted experiments with various doping concentrations ranging from 0% to 8% and particle sizes of 132 μm, 180 μm, and 230 μm. Their research uncovered that an increase in both the particle size of the microencapsulation and the degree of doping resulted in a drop in compressive strength.

**Figure 34:**
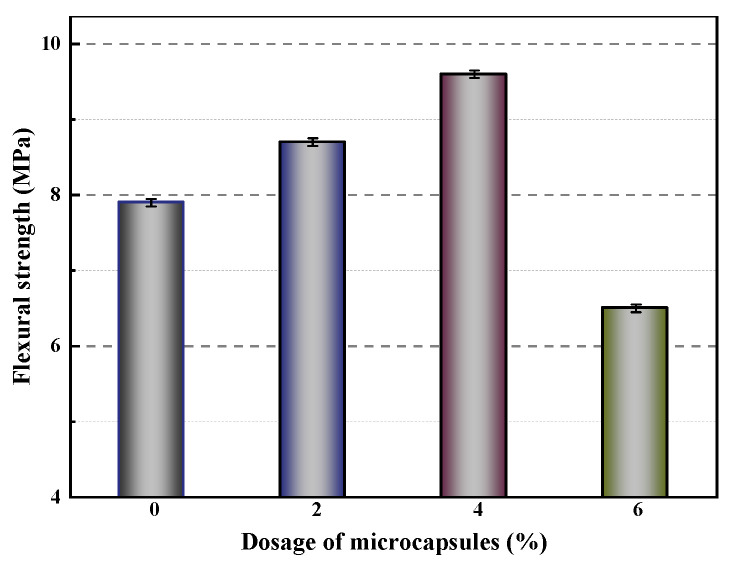
Flexural strength of mortars containing microcapsules [75].

Figure 34 illustrates the variation in flexural strength of mortar with different microcapsule contents after 28 days. The results show that flexural strength increases initially with the addition of microcapsules but then decreases as the microcapsule content rises further. This trend is due to the significant difference in Young’s modulus between the microcapsules and the cement-based matrix, coupled with gaps in the bonding surfaces between the microcapsules and the matrix material. A moderate amount of microcapsules enhances the compactness of the mortar, leading to a denser structure and increased flexural strength. However, increasing the microcapsule content to 6% results in a 17.7% reduction in flexural strength.

**Figure 35:**
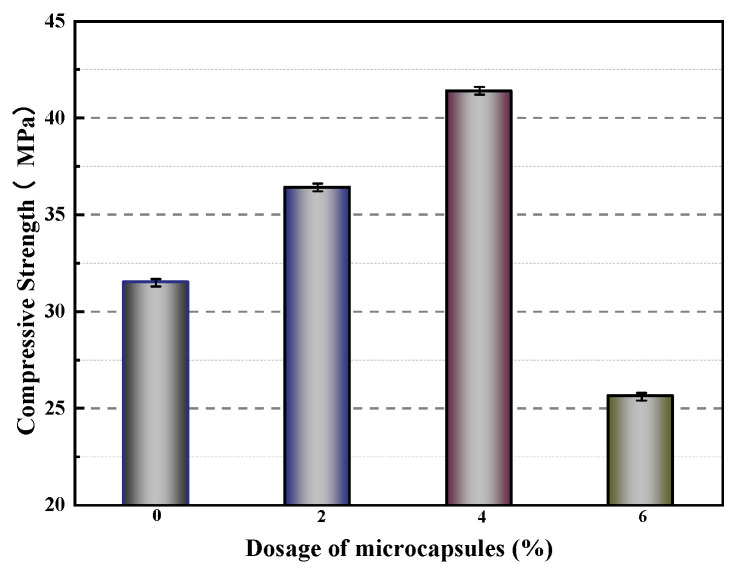
Compressive strength of mortars containing microcapsules [75].

Figure 35 depicts the impact of varying microcapsule contents on the compressive strength of mortar. The compressive strength of M-1 (microcrystalline wax/epoxy resin microcapsules mixed with 2% of the cement mass) and M-2 (microcrystalline wax/epoxy resin microcapsules mixed with 4% of the cement mass) increased by 15.6% and 31.4%, respectively, compared to M-0 (control concrete). This improvement is attributed to the optimal amount of microcapsules filling internal voids within the mortar, thereby enhancing its compressive strength. Conversely, M-3 (microcrystalline wax/epoxy resin microcapsules mixed with 6% of the cement mass) shows a 5.7% decrease in compressive strength. This reduction is due to the large disparity in modulus between the microcapsules and the cement matrix, which creates voids at the bonding surfaces and decreases the compactness and particle gradation of mortar, ultimately diminishing compressive strength.

**Figure 36:**
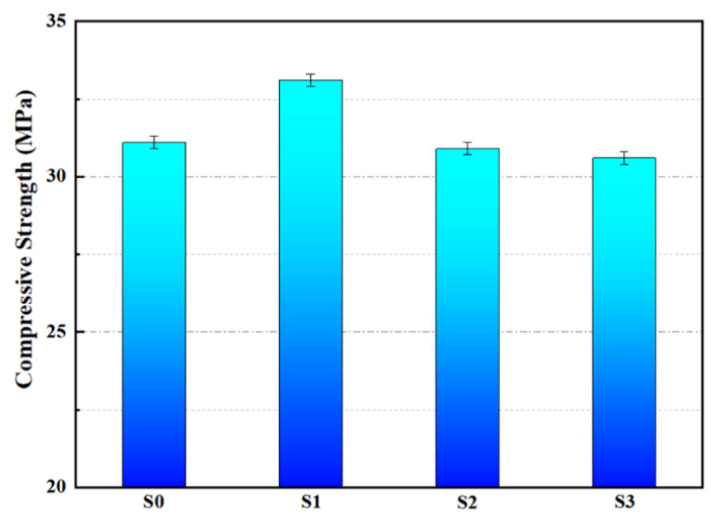
Compressive strengths of S0–S3 [48].

**Figure 37:**
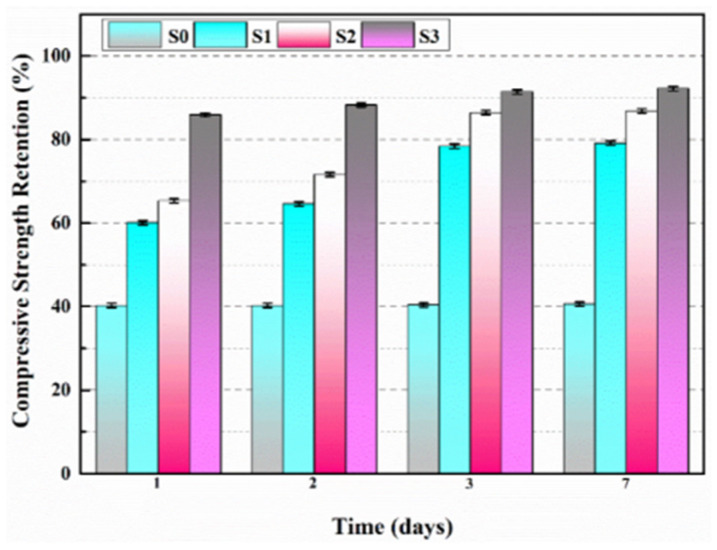
The compressive strength retention of S0–S3 [48].

Figure 36 presents the compressive strength of mortar specimens after 28 days of curing. S0 had a compressive strength of 31.1 MPa, while S1 achieved 33.1 MPa, S2 had 30.9 MPa, and S3 reached 30.6 MPa. The compressive strength of S1 increased by 6.4%, whereas S2 and S3 exhibited slight decreases in strength. The improved compressive strength in S1 is attributed to the small particle size of microencapsulated M1, which acts as a fine aggregate that effectively distributes stress. Additionally, the porous structure of the mortar allows M1 to fill in the pores, enhancing compactness and increasing compressive strength. In contrast, M2 and M3, with their larger particle sizes, are less effective in filling the micropores, resulting in a minimal impact on the compressive strength of S2 and S3, as illustrated in Figure 37.

The interaction between microcapsules and the cement matrix significantly influences flexural strength. Key factors include the chemical composition of the microcapsules, their dispersion within the cement, and the nature of the physical and chemical interactions at the interface. Reactive or incompatible materials within the microcapsules can result in weak bonding at the interface, thereby affecting the overall mechanical properties of the cement matrix. Uniform dispersion of microcapsules is essential, as clustering or aggregation can create localized stress concentrations, which may diminish the flexural strength of material. Additionally, the size and shape of the microcapsules impact their behavior within the cement matrix. Physical and chemical interactions—such as hydrogen bonding, ion exchange and adsorption, and cross-linking—can affect the performance of material by causing increased brittleness or embrittlement. Thus, careful consideration of these factors is vital when designing and implementing cement matrices with microcapsules to ensure that the final product meets the desired performance standards [76,77,78,79].

The incorporation of excess microcapsules influences the mechanical properties of cement-based materials, which is due to several factors. (1) In the filling effect, high microcapsule addition occupies space, reducing cement paste volume. This diminishes concrete compactness, affecting strength. Additionally, high concentrations may alter pore structure, increasing porosity and negatively impacting strength [80]. (2) In chemical reactions, microcapsules often contain chemicals or phase change materials that undergo reactions or changes under heat or other conditions. These can affect concrete microstructure and mechanical properties. For instance, phase-change materials can cause volume changes when absorbing/releasing heat, causing microscopic damage. (3) In microstructure changes, microcapsules can react with the cement matrix to form new mineral phases like C-S-H, improving densification and strength. However, discontinuities form if there is incomplete encapsulation or uneven dispersion, becoming a starting point for crack expansion and reducing strength. (4) In hydration process effects, encapsulation properties can hinder water penetration into microcapsule interiors, resulting in incomplete hydration reactions around microcapsules and affecting hardening and strength development overall.

2. Permeability

Permeability and durability are closely linked in concrete, an inherently porous and absorbent material that can absorb liquids. Key characteristics of concrete, such as pore volume, pore structure, and permeability, are crucial in determining its performance. Concrete permeability affects how easily gases, liquids, and soluble hazardous chemicals can penetrate the material. This directly impacts carbonation, erosion, steel reinforcement corrosion, and freeze–thaw durability, making it a critical factor influencing the longevity of concrete structures. The permeability of concrete can change due to micro-cracks and variations in pore structure, which can, in turn, affect the durability of material through its self-repairing capabilities. Therefore, assessing the permeability of cement-based self-repairing materials is vital for evaluating their repair effectiveness. There are a variety of methods that can be used to assess the durability and permeability of concrete. Among them, the Rapid Chloride Penetration (RCP) test, although complex, is a standard procedure for evaluating the resistance of concrete to chloride ion penetration [81].

**Figure 38:**
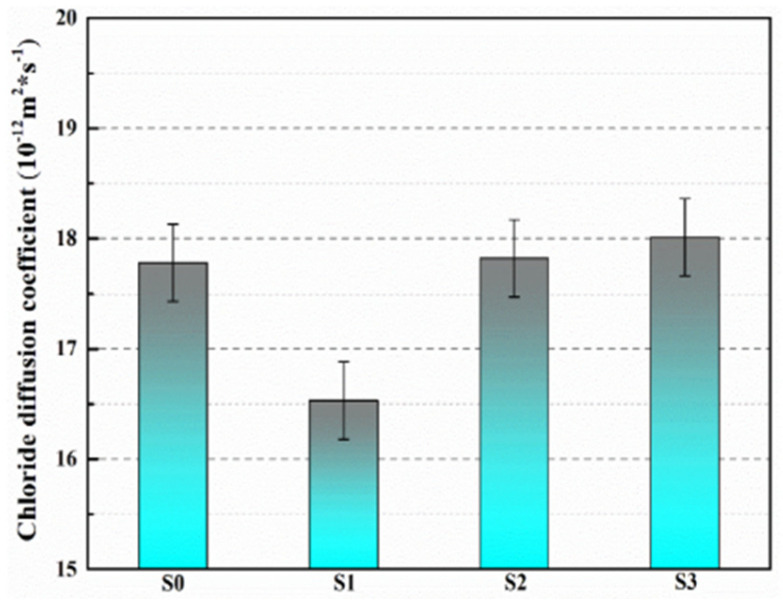
Chloride diffusion coefficient of S0–S3 [48].

**Figure 39:**
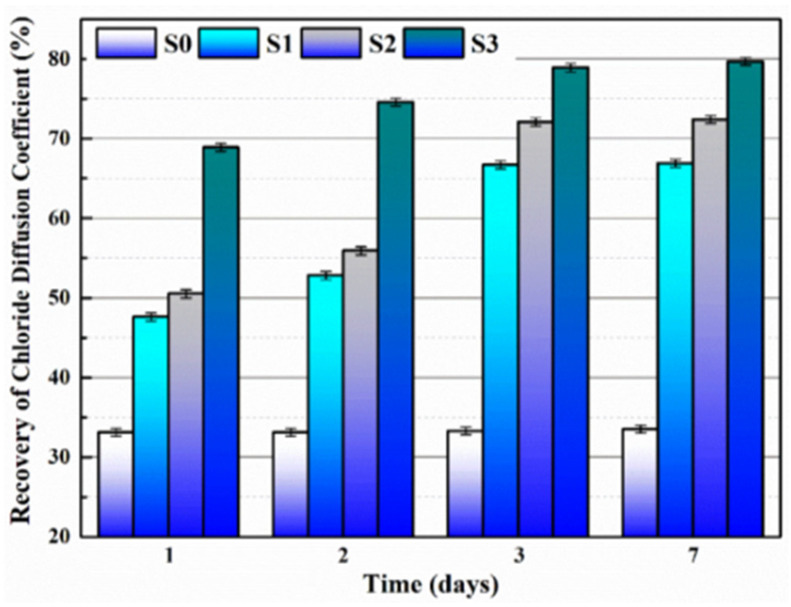
The recovery of the chloride diffusion coefficient of S0–S3 after self-repairing [48].

The densification of the mortar is assessed using the chloride diffusion coefficient in Figure 38, illustrating the link between microcapsule filling action and the mortar. S1 exhibited the lowest chloride ion diffusion coefficient, suggesting that it was filled with the best M1 microcapsules with the highest degree of densification, according to a comparison of the chloride ion diffusion coefficients of S0, S1, S2, and S3 at 28 days. Due to higher particle sizes, which decreased the density of mortar and decreased its impermeability, M2 and M3 were filled less than optimally. The chloride diffusion coefficient in the mortar containing microcapsules was restored, as depicted in Figure 39. After seven days of self-repairing, the chloride diffusion coefficient of S0 did not recover even when the preload was 80% f_0_. This indicates that the damaged structure within the mortar cannot be repaired using only cement-based material. For 24 h spanning two to three days of self-repair, the chloride diffusion coefficients for S1 and S2 showed a significant increase in recovery, reaching 66.7% and 72.1%, respectively. M2 is more efficient at self-repairing mortar than M1 due to its higher core composition. The M3 shell material generates heat due to the magnetic material, leading to the melting and rupture of the microcapsule. As a result, S3 demonstrates a faster recovery of chloride diffusion coefficients compared to S1 and S2. At elevated temperatures, the reaction between IPDI and moisture intensifies, leading to the rapid production of additional polyurea, effectively enhancing the impermeability of the mortar.

**Figure 40:**
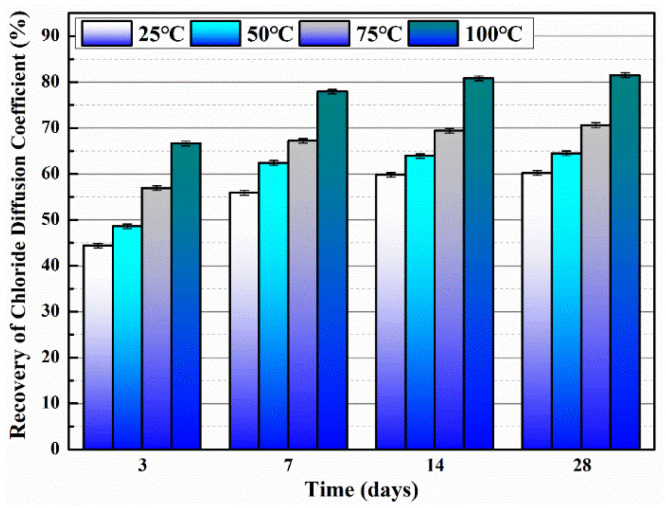
Recovery of the chloride diffusion coefficient of CA1 at various temperatures [58], Copyright, 2023, Elsevier, License Number 5774050316089.

**Figure 41:**
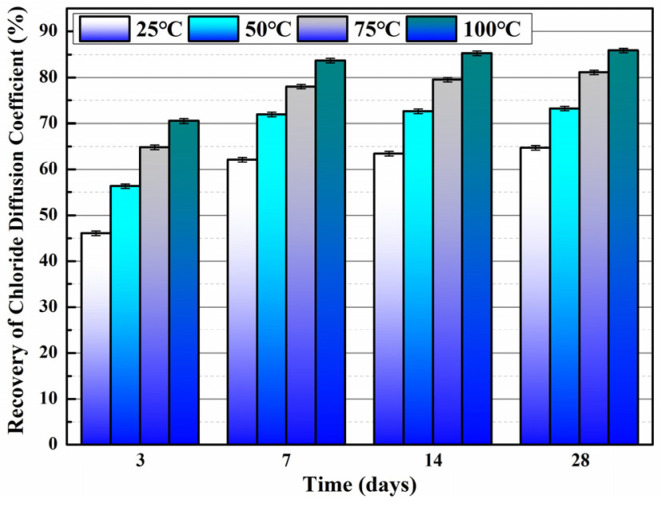
Recovery of the chloride diffusion coefficient of CA3 under various temperatures [58], Copyright, 2023, Elsevier, License Number 5774050316089.

Figure 40 and Figure 41 display the restoration of the chloride diffusion coefficients of CA1 (OPC mortar containing microcapsules) and CA3 (LC3 mortar containing microcapsules) at various temperatures. The chloride diffusion coefficients of CA1 and CA3 recovered more quickly during 3–7 days of self-repair but stabilized after 14 days. Microcracks inside CA1 and CA3 facilitated chloride ion transport during preloading, increasing chloride ion diffusion coefficient and reducing permeability resistance. The microcapsules were included in CA1 and CA3 during their production. The microcapsule shells broke when internal cracks formed under external loading. The rupture allows the epoxy resin and 2-ethyl-4-methylimidazole reaction to fill the cracks, enhancing the internal densification of CA1 and CA3. This process reduces the chloride ion diffusion coefficient and restores impermeability. Microcapsules significantly improved the recovery of the chloride ion transport coefficient of CA1 and CA3 at higher temperatures.

The RCP test can be completed in a short period of time compared to the traditional long-term immersion test, which greatly saves time and cost. Studies show that the tendency of chloride ion penetration through cracks decreases with time, especially in samples with large crack widths [82]. In addition, the RCP test can also evaluate the effect of different admixtures on the chloride penetration performance of concrete, providing a basis for the optimization of concrete materials. However, the RCP test also has some limitations, such as the sensitivity to certain admixtures and additives that may lead to biased results. Future research may focus on improving the accuracy and reliability of RCP tests. For example, by improving the testing methods and equipment, the errors and biases in the testing process can be reduced. Meanwhile, with the development of new materials and technologies, how to incorporate these new materials and technologies into RCP testing to more accurately assess the self-repairing ability and durability of concrete will be an important direction for future research.

In summary, the long-term effects of altered permeability on the chemical resistance of cement are complex and involve several factors, including changes in the permeability coefficient in acidic environments, the use of chemical additives and microfilters, and the application of curing materials. These elements collectively influence the chemical resistance and overall durability of cement under varying environmental conditions.

3. Ultrasonic testing

Ultrasound is a non-destructive testing method that evaluates the extent and quality of damage within a material. Fast Fourier Transform (FFT), a mathematical process, is used to analyze the spectrum of ultrasonic waves, enabling the identification of material properties and structural variations. FFT can assess the successful integration and dispersion of microcapsules within the material by examining ultrasonic signals. This non-destructive approach allows for the effective incorporation of microcapsule self-repairing technology with a reliable quality control method.

**Figure 42:**
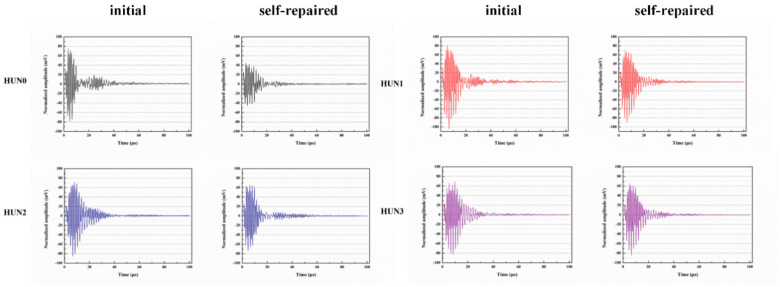
The ultrasonic waveform of concretes [56], Copyright, 2021, Elsevier, License Number 5774040151380.

**Figure 43:**
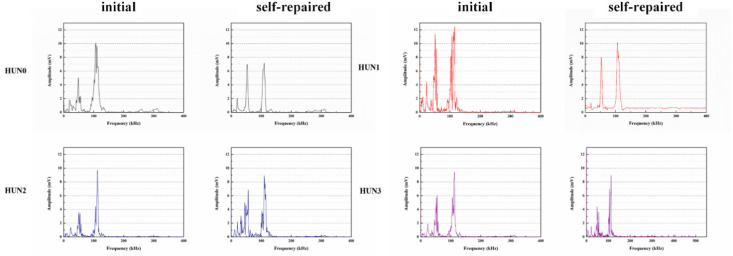
Ultrasonic frequency of concretes [56], Copyright, 2021, Elsevier, License Number 5774040151380.

Figure 42 illustrates the ultrasonic signals of various concrete samples following self-repair. The results reveal that concrete samples containing microcapsules (HUN1, HUN2, and HUN3) showed improved recovery of maximum amplitude after self-repair, with HUN3 achieving the amplitude closest to the initial value. This suggests that when microcracks formed in the concrete, the microcapsule shells ruptured under stress, releasing low-viscosity, flowable IPDI. This IPDI reacted with water in the concrete to form healing products that filled the microcracks, reducing internal defects and enhancing concrete densification and amplitude. Figure 43 presents the ultrasonic frequency data for different concrete samples post repair. The results demonstrate that concrete samples with microcapsules experienced a recovery in maximum amplitude after self-repair, with the amplitude of HUN3 approaching to the initial value. This indicates that microcapsules effectively reduce internal defects, improve concrete compactness, and enhance the maximum amplitude of the ultrasonic signal. Specific data show that the maximum amplitude for the control group (HUN0) is 44.15 mV, while HUN1, HUN2, and HUN3 have maximum amplitudes of 70.23 mV, 64.93 mV, and 64.74 mV, respectively. These findings suggest that the addition of IPDI microcapsules improves the sulfate resistance and self-repairing properties of concrete and confirms their efficacy in repairing concrete structural damage.

**Figure 44:**
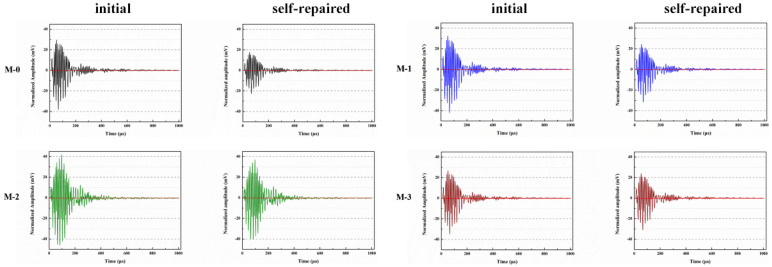
Ultrasonic waveform of mortars with microcapsules before and after self-repair [75].

**Figure 45:**
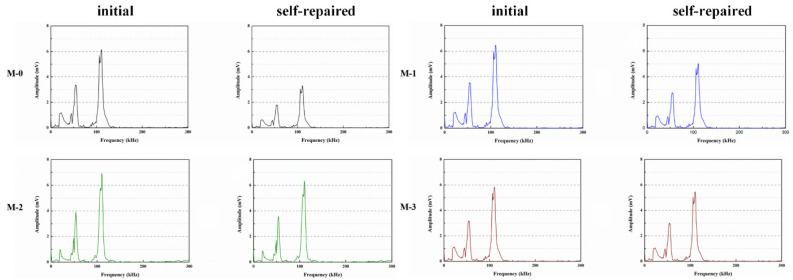
Ultrasonic frequency of mortars with microcapsules before and after self-repair [75].

Figure 44 displays ultrasonic waveforms for cement mortars containing varying amounts of microcapsules before and after self-repair. After 28 days of standard curing, the maximum wave amplitudes for M-0, M-1, M-2, and M-3 were 29.63 mV, 32.59 mV, 42.15 mV, and 26.93 mV, respectively. Compared to M-0, the amplitudes for M-1 and M-2 increased, while M-3 decreased. This variation is primarily due to ultrasonic wave attenuation caused by pores in the cement mortar. Denser mortar with fewer internal defects results in less attenuation and relatively higher wave amplitudes. After 14 days of self-repair, the maximum wave amplitudes for M-0, M-1, M-2, and M-3 were 17.51 mV, 24.44 mV, 37.09 mV, and 23.97 mV, respectively. This indicates significant recovery of wave amplitude in mortars containing microcapsules. The improvement is attributed to the rupture of microcapsules under crack tip stress, which releases E-51 epoxy resin mixed with 2-ethyl-4-methylimidazole to fill microcracks and pores, thereby enhancing mortar densification and wave amplitude. Figure 45 shows the changes in ultrasonic frequency for mortars with different microcapsule contents before and after self-repair. The data from 28 days of standard maintenance and 14 days of self-repair show similar trends. Specifically, the maximum amplitudes of M-0, M-1, M-2, and M-3 were 17.51 mV, 24.44 mV, 37.09 mV, and 23.97 mV, respectively, after 14 days of self-repair. The significant restoration of maximum amplitudes in microcapsule-containing mortars suggests that E-51 epoxy resin released from ruptured microcapsules effectively cured and filled microcracks and pores, improving mortar densification. In summary, the data from Figure 35 and Figure 36 confirm the efficacy of microcapsules in self-repairing cement-based materials, particularly in enhancing mortar densification and reducing internal defects.

## 4. Prospects and Summaries

The development of self-repairing cement-based materials represents a transformative advancement in construction technology, offering the potential to significantly extend the lifespan of structures by enabling the autonomous repair of internal micro-cracks and defects. This innovation addresses one of the most pervasive challenges in the construction industry—crack-related damage—while providing a potential solution for reducing the high maintenance and replacement costs associated with traditional repair methods. The self-repairing mechanism is especially crucial in mitigating the deterioration of concrete structures, which are subjected to a wide array of environmental stressors, such as thermal fluctuations, moisture, and chemical attacks. This technology offers a promising solution for both enhancing the durability of structures and lowering lifecycle costs, making it particularly attractive for large-scale construction projects.

A variety of self-repair techniques for cement-based materials have been explored, including hollow fiber technology, microbial self-repair systems, shape-memory alloys, and osmotic crystallization. Among these, microcapsule-based self-repair technology stands out due to its superior adaptability, rapid healing capabilities, and potential for integration with existing construction materials. The concept behind microcapsule self-repair technology is based on encapsulating one or more healing agents within micro-sized capsules, which are integrated into the cement matrix. Upon the formation of cracks or defects, the microcapsules rupture, releasing the healing agents into the damaged areas. These agents then react with other cement constituents, forming new material structures that effectively seal the cracks and restore the integrity of the concrete. This system is highly advantageous due to several key characteristics. (I) Wide applicability: Microcapsules can be utilized with various cement-based materials and exhibit strong performance across different environmental conditions. (II) Fast healing: The release of healing agents occurs immediately upon crack formation, enabling swift and efficient self-repair. (III) High controllability: The size, quantity, and distribution of microcapsules can be tailored to regulate the self-repair process. Despite these advantages, the technology is challenged by limited large-scale applications and issues related to mechanical integrity.

Despite these advantages, microcapsule-based self-repair technology faces several challenges, particularly when considering its large-scale application. One of the primary limitations is the high cost of manufacturing microcapsules, as the encapsulation process typically involves intricate chemical methods that can be expensive. However, the long-term economic benefits—such as reduced maintenance, fewer repairs, and extended material lifespan—could outweigh the initial costs. Additionally, mechanical integrity remains a concern, as the microcapsules themselves must be strong enough to withstand the stresses of mixing and placement within the concrete without prematurely rupturing.

When it comes to large-scale implementation, challenges such as uniform distribution of microcapsules during mixing, stability of the capsules in storage and during construction, and the adaptation of existing construction methods to accommodate microencapsulation must be addressed. Ensuring the stability of microcapsules over time, especially during long-term storage and exposure to varying environmental conditions, is crucial for maintaining their effectiveness. Furthermore, there are specific construction techniques that must be refined to ensure the consistent integration of microcapsules into the mix and their reliable activation when cracks form.

To overcome some of these challenges, recent research has focused on reinforcing the microcapsule shells with nanomaterials such as silica, carbon nanotubes, and magnetic nanoparticles to enhance the mechanical strength and durability of the capsules. This innovative approach increases the resilience of the capsules, ensuring they remain intact until the onset of crack formation, and improves their ability to withstand mechanical stresses during handling and placement. Additionally, the incorporation of magnetic nanoparticles enables a more controlled activation of the self-repair process, potentially offering a way to direct the healing agents to specific locations within the structure.

In terms of application, microcapsule self-repair technology has the potential to revolutionize maintenance practices in the construction industry. By integrating this technology into concrete used in bridges, tunnels, roads, and high-rise buildings, the need for frequent inspections and repairs could be drastically reduced. Moreover, the technology offers eco-friendly benefits by extending the life of existing structures, reducing the need for replacement materials, and lowering the overall carbon footprint associated with construction and demolition.

The present research contributes significantly to the literature by expanding the understanding of microcapsule-based self-repair mechanisms, identifying the limitations that need to be addressed for large-scale implementation, and providing insight into how various reinforcement strategies can improve capsule performance. Future work in this field should focus on several key areas. (I) Improved encapsulation techniques: Advancements in encapsulation materials and processes can improve the cost-effectiveness and scalability of microcapsules, making them more suitable for large-scale projects. (II) Long-term performance studies: Field trials and long-term monitoring are essential for evaluating the actual effectiveness and durability of microcapsule self-repair systems in real-world conditions. (III) Integration with digital technologies: The development of smart concrete systems, which combine microcapsule technology with sensors and real-time monitoring, could provide additional insights into the performance of self-healing materials, offering an automated and proactive approach to structural maintenance. (IV) Eco-friendly materials: The incorporation of sustainable, biodegradable, or recycled materials in the fabrication of microcapsules and their shell materials could further enhance the environmental benefits of this technology. (V) Multidisciplinary integration: Future research could explore integrating microcapsule-based self-repair with other advanced repair technologies, such as shape-memory alloys or microbial systems, to create hybrid self-healing systems that can address a broader range of structural damage.

In conclusion, microcapsule self-repair technology for cement-based materials represents a promising frontier in construction materials innovation. While several challenges remain, continued research and development, particularly in the areas of cost reduction, performance enhancement, and large-scale integration, hold the key to unlocking the full potential of this technology. By pushing the boundaries of materials science and engineering, microcapsule-based self-repair could lead to a paradigm shift in how we approach construction and maintenance, providing sustainable, cost-effective solutions for the future of the built environment.

## Figures and Tables

**Table 1:** Summary of shell materials and healing materials.

Preparation Methods	Shell Material	Healing Agent	Reported Major Findings	References
Glass tube encapsulation method	Gelatin	Acrylic resin	The reduced presence of the healing agent within the capsules leads to diminished healing efficacy.	[42]
Interfacial self-assembly and sol–gel reactions	Silica gel	Methylmethacrylate	A decrease in permeability leads to an enhancement of the self-healing capability in mortar.	[4]
In situ polymerization	Polyurethane	Sodium Silicate	The addition of microcapsules not only enhances the flexural strength but also markedly suppresses corrosion, as observed.	[43]
In situ polymerization	Urea-formaldehyde	Epoxy resin	Microcapsules characterized by excellent surface texture, appropriate dimensions, robustness, and notable thermal stability are manufactured, yielding high rates of crack repair and effectively countering chloride ingress.	[44]
In situ polymerization	Melamine urea formaldehyde	Epoxy resin	Various factors affect the preparation of microcapsules, i.e., stirring rate, pH, core-wall ratio, and temperature.	[45]
In situ polymerization	Polyurethane/urea formaldehyde	Sodium Silicate	Microcapsules with a double-walled structure of PU/UF exhibit enhanced durability when compared to their single-walled counterparts.	[46]
Melting dispersion condensation method	Fe_3_O_4_ nano-particles/polyethylene wax	epoxy resin	Electromagnetic-induced rupture microcapsules effectively enhance the self-repairing ability of cement-based materials.	[47]
Melting dispersion condensation method	Polyethylene wax/ferrous powder	IPDI	Electromagnetic inductive microcapsules enhance the mechanical properties and self-repairing ability of LC3 mortar.	[48]
